# Chemical approaches to discover the full potential of peptide nucleic acids in biomedical applications

**DOI:** 10.3762/bjoc.17.116

**Published:** 2021-07-19

**Authors:** Nikita Brodyagin, Martins Katkevics, Venubabu Kotikam, Christopher A Ryan, Eriks Rozners

**Affiliations:** 1 Department of Chemistry, Binghamton University, The State University of New York, Binghamton, New York 13902, United States; 2 Latvian Institute of Organic Synthesis, Aizkraukles 21, Riga, LV-1006, Latvia

**Keywords:** antisense, chemical modifications, diagnostics, peptide nucleic acid, PNA

## Abstract

Peptide nucleic acid (PNA) is arguably one of the most successful DNA mimics, despite a most dramatic departure from the native structure of DNA. The present review summarizes 30 years of research on PNA’s chemistry, optimization of structure and function, applications as probes and diagnostics, and attempts to develop new PNA therapeutics. The discussion starts with a brief review of PNA’s binding modes and structural features, followed by the most impactful chemical modifications, PNA enabled assays and diagnostics, and discussion of the current state of development of PNA therapeutics. While many modifications have improved on PNA’s binding affinity and specificity, solubility and other biophysical properties, the original PNA is still most frequently used in diagnostic and other in vitro applications. Development of therapeutics and other in vivo applications of PNA has notably lagged behind and is still limited by insufficient bioavailability and difficulties with tissue specific delivery. Relatively high doses are required to overcome poor cellular uptake and endosomal entrapment, which increases the risk of toxicity. These limitations remain unsolved problems waiting for innovative chemistry and biology to unlock the full potential of PNA in biomedical applications.

## Introduction

**Figure 1:**
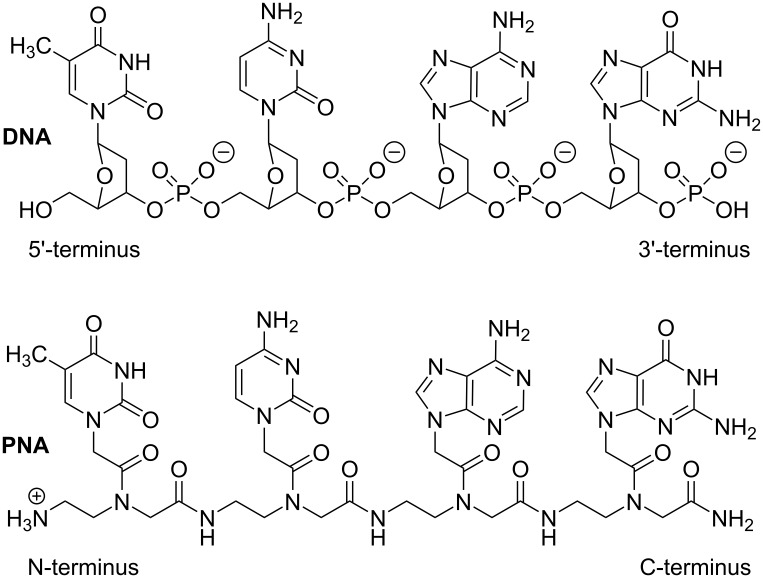
Structure of DNA and PNA.

Peptide nucleic acid (PNA) is a DNA mimic where the sugar–phosphate backbone of DNA is replaced with a neutral and achiral pseudopeptide backbone (Figure 1) [1]. PNA retains the natural DNA nucleobases that are connected to the amide-linked backbone through additional amide linkages. PNA was originally designed as a DNA mimic to improve the properties of triplex-forming oligonucleotides [1–2]. Two key considerations were elimination of electrostatic repulsion (neutral backbone) and synthetic accessibility (simple to make achiral amide linkages) [3]. The design was guided by a simple computer model where the natural sugar-phosphodiester backbone of the Hoogsteen strand of a T•A–T DNA triplex was replaced by an achiral and neutral pseudopeptide backbone having the same number of atoms [2–3]. It is remarkable that this simple design resulted in a nucleic acid analogue that had the right degree of flexibility and favorable conformational properties, enforced by the rotational preferences around amide linkages, to form strong and sequence specific complexes with natural DNA and RNA [3]. As will be discussed below, despite extensive studies [4–6], relatively few modifications have improved this simple original design.

Since its inception, PNA has become an extremely useful research tool and enabling component of many assays and diagnostics [4,7–9]. On the other hand, development of PNA based therapeutics has notably lagged behind other nucleic acid technologies [10–11]. In the present review, we summarize the remarkable journey of PNA from the initial design, through many chemical modifications and various applications, to the current state of the field. We also seek insights into the key question of why PNA, despite its impressive biophysical properties, has still not entered clinical trials.

The most significant difference between PNA and the natural nucleic acids is the lack of negative charge on PNA’s backbone. Electrostatic repulsion of the negatively charged phosphates dominates the conformational properties and structure of nucleic acids. In contrast to proteins that prefer to fold in compact structures, DNA and RNA inherently prefer extended conformations that minimize the electrostatic repulsion. The maintenance and function of long double-stranded DNA (dsDNA) is achieved through complex mechanisms involving histones and other proteins. Large non-coding RNAs (e.g., ribosomes) manage electrostatic repulsion using positively charged RNA-binding proteins and cations (e.g., magnesium ions), and achieve remarkably complex folded structures. Nevertheless, the electrostatic repulsion is the main force that disfavors folding and association of nucleic acids. With this consideration in mind, neutral PNA was expected to have superior binding to negatively charged nucleic acids due to the lack of electrostatic repulsion [1–3].

As will be reviewed below, because of its robust metabolic stability and high affinity and sequence specificity, PNA has become a vital component of many research assays and diagnostics [4]. Nevertheless, PNA has not been without shortcomings and vulnerabilities. Limited water solubility, especially for purine rich sequences, was noted in early studies. To improve water solubility and decrease aggregation, typical PNA designs place a lysine at the C-terminus (Figure 1) introducing a second positive charge in addition to the charge at the N-terminus of PNA [1]. Even with the additional lysine, the solubility of PNA decreases as the polymer length increases. PNA solubility in the HEPES buffer at pH 7.3 and 37 °C is estimated to be in the 0.1–0.5 mM range [12–13]. The hydrophobic nature and lack of electrostatic repulsion of the PNA backbone favors folding in compact structures and aggregation in concentrated solutions [13].

Other bottlenecks for in vivo applications of PNA have been poor cellular uptake and unfavorable pharmacokinetics [14–16]. Unmodified PNAs are not taken up by eukaryotic cells in vitro and are cleared rapidly (within 10–30 min in mice) through the kidneys after administration to animals by either intravenous or intraperitoneal injection [16]. In another study, PNA elimination half-life in rats was ≈17 minutes and ≈90% of PNA was recovered unchanged in the urine 24 h after administration [17].

To address these problems, many research groups have worked on chemical modifications to the backbone and nucleobases of PNA, as well as conjugating PNA to other biomolecules (e.g., cell-penetrating peptides) [4]. The present review summarizes the most significant efforts and achievements in optimizing various aspects of PNA applications. We start with a brief review of PNA’s binding modes and structural features, continue to the most impactful chemical modifications, PNA enabled assays and diagnostics, and finish with discussion of the current state of development of PNA therapeutics. The common theme that emerges is that despite extensive studies reviewed below, PNA still needs innovative chemistry to break through in clinic and other in vivo applications.

## Review

### PNA binding modes to DNA and RNA

**Figure 2:**
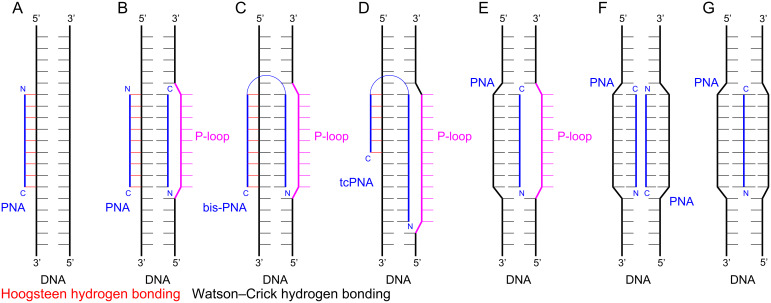
PNA binding modes: (A) PNA–dsDNA 1:1 triplex; (B) PNA–DNA–PNA strand-invasion triplex; (C) the Hoogsteen and Watson–Crick parts are linked together in a bis-PNA; (D) shortening the Hoogsteen part and extending the Watson–Crick part of the bis-PNA creates a tail-clamp PNA (tcPNA); (E) and (F) single and double invasion using only Watson–Crick hydrogen bonding; (G) Janus-wedge triple helix.

PNA was originally designed with an expectation to improve the binding properties of negatively charged triplex-forming oligonucleotides to dsDNA [1–2]. The parallel PNA-dsDNA triplex, where the N-terminus of PNA aligns with the 5′-terminus of a polypurine strand of DNA (Figure 2A), is a binding mode that is particularly sensitive to electrostatic repulsion as three negatively charged strands are brought in proximity. PNA was also found to bind single-stranded DNA and RNA (ssDNA and ssRNA) in an antiparallel fashion (the C-terminus of PNA aligning with the 5′-terminus of ssDNA) with affinity and sequence selectivity significantly higher than that of the natural oligonucleotides [18–19]. The thermal stabilities of duplexes involving PNA usually follow the order PNA–PNA > PNA–RNA > PNA–DNA [20–21]. Hybridization of PNA with complementary nucleic acids is enthalpy driven, involving large favorable gains in enthalpy compensated by significant unfavorable entropy, as typically observed for nucleic acid complexes [22]. The binding is highly sequence specific as one Watson–Crick base pair mismatch can drop the melting temperature of the complex with PNA by 8–20 °C making PNA an excellent nucleic acid analogue for development of probes and diagnostics. This strong and selective binding has made PNA a key component of assays and diagnostics that depend on Watson–Crick hydrogen bonding to natural nucleic acids. An unexpected discovery of early studies was that the triplex-forming PNAs built of pyrimidine monomers formed a 2:1 PNA–DNA–PNA strand-invasion triplex instead of the expected 1:1 PNA–dsDNA triplex (c.f., Figure 2A and 2B) [1,23]. This unprecedented binding mode was enabled by PNA’s unique ability to displace the pyrimidine-rich strand of dsDNA as the so-called P-loop, which was clearly facilitated by the neutral backbone [1].

Later studies showed that there was a delicate balance between the two binding modes. The strand invasion (Figure 2B) was favored at low ionic strength and high PNA concentration, and required longer reaction times [24]. In contrast, physiological ionic strength inhibited strand invasion and shifted the binding mode towards the major groove Hoogsteen triple helix (Figure 2A) [24]. The binding mode was also affected by PNA’s sequence with thymine-rich PNAs generally preferring invasion complexes and cytosine-rich PNAs generally preferring triple helix formation [25]. Overall, while PNA formed stronger triple helices with dsDNA than negatively charged oligonucleotides, the stability of the triplexes was still lower than that of the Watson–Crick PNA–DNA and PNA–RNA duplexes and required a tract of at least 15 consecutive purines for chemically-modified triplex-forming PNA to achieve low nanomolar binding [26]. Triple-helical binding of PNA to dsRNA was not explored until 2010 when Rozners and co-workers showed that PNAs as short as hexamers formed strong and sequence specific triplexes at pH 5.5 [27]. Later studies using nucleobase-modified PNA (vide infra) confirmed that PNA had >10-fold higher affinity for dsRNA than for the same sequence of dsDNA [28–31].

While parallel PNA–DNA and PNA–RNA triple helices formed by PNAs built of C and T monomers are well documented (as reviewed above), the antiparallel triplexes formed by PNAs built of G and T or G and A monomers have not been reported. It is conceivable, that the limited solubility and tendency to aggregate prevent such binding modes involving purine-rich PNAs, as discussed in a recent review [32]. However, it is also possible that this is an underexplored PNA binding mode. G-rich PNAs do not form stable G-quadruplexes [33], which suggests that with innovative chemistry, it may be possible to explore G-rich PNAs for antiparallel triplexes.

The strand invasion complex contains two PNA molecules binding the purine-rich strand of DNA. While one PNA strand forms an antiparallel Watson–Crick duplex, the other strand forms a parallel Hoogsteen triplex, which brings the N- and C-ends of the two strands in proximity (Figure 2B). An innovative design links the two ends together with an ethylene glycol linker (Figure 2C), which reduced the unfavorable loss of entropy by converting the binding event from a trimolecular to a bimolecular process [34–36]. The new bis-PNAs (Figure 2C) showed about two orders of magnitude stronger binding (lower EC_50_) to ssDNA targets compared to the trimolecular formation of the PNA–DNA–PNA triplex [35]. However, the need for polypurine tracts remained a limitation of bis-PNAs. A further development that extended the sequence scope that can be targeted by bis-PNAs was to shorten the Hoogsteen part and extend the Watson–Crick part of the bis-PNA by creating a tail-clamp PNA (tcPNA, Figure 2D) [37]. Tail-clamp PNAs are currently at the forefront of PNA therapeutic development (vide infra).

Single or double invasion of dsDNA (Figure 2E and 2F, respectively) using only Watson–Crick base pairing at mixed sequences that do not have polypurine tracts is also possible, but requires chemical modifications to alter the binding properties of PNAs. These binding modes further illustrate the diversity of molecular recognition that can be achieved with PNAs. Taken together, the early discoveries that revealed the remarkable nucleic acid binding properties of PNA boosted enthusiasm about PNA’s potential as an antisense and antigene therapeutic agent [38].

### Structures of PNA complexes

Early NMR structural studies suggested that PNA formed heteroduplexes with DNA [39] and RNA [40] that resembled the B- and A-form conformations of natural nucleic acids. The PNA–RNA duplex adopted a conformation very close to the standard A-form helix [40]. In contrast, the PNA–DNA duplex adopted an intermediate structure where positioning of the base pairs was A-like, while the backbone curvature, sugar conformation (C2′-endo), base pair inclination, and helical rise resembled B-DNA [39].

**Figure 3:**
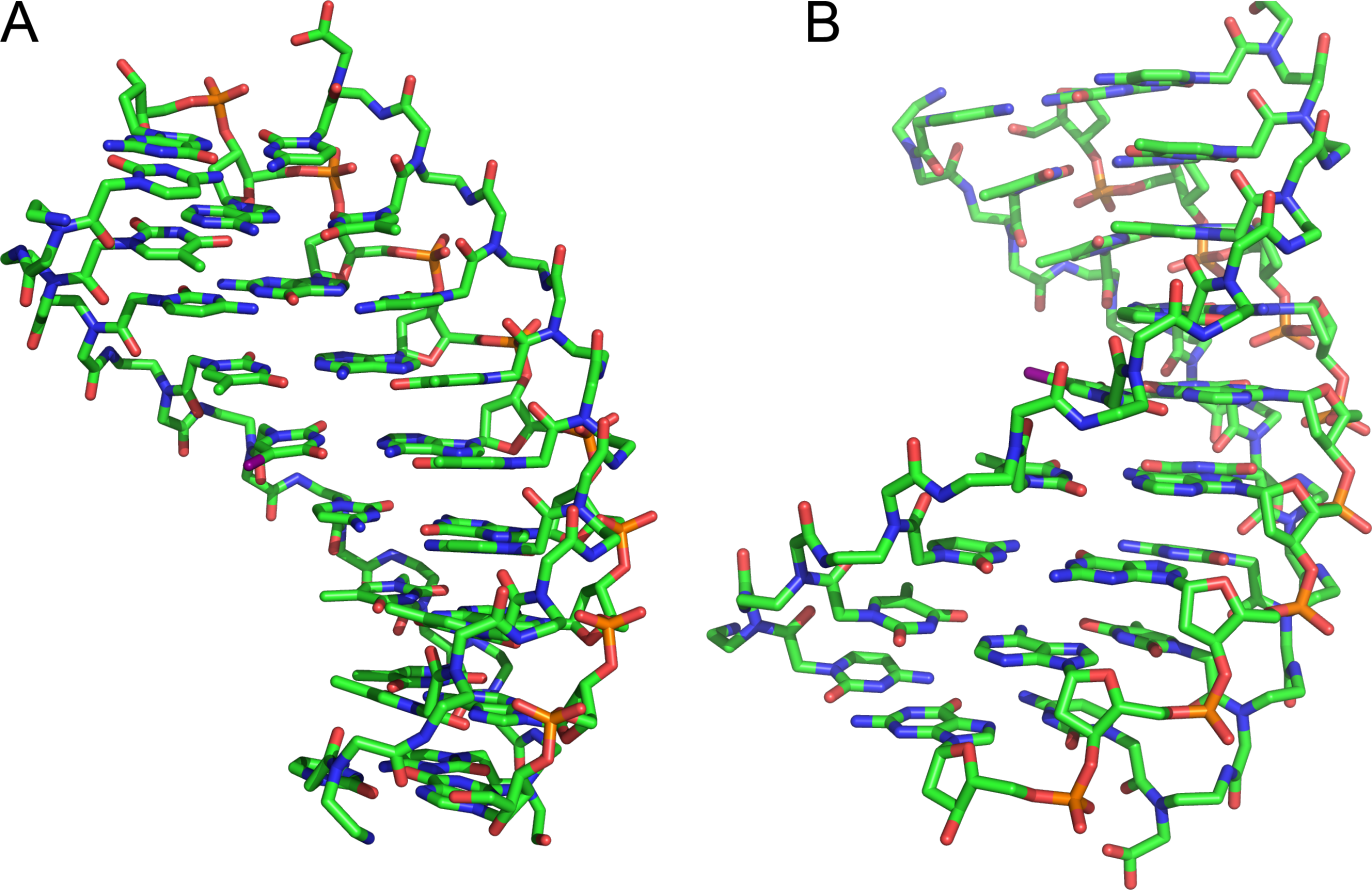
Structure of P-form PNA–DNA–PNA triplex from reference [41]. (A) view in the major groove and (B) view in the minor groove.

The first X-ray crystal structure of a PNA–DNA–PNA triplex revealed a previously unknown helix with a wide diameter of ≈26 Å (compared to 20 Å for A-form duplex) and a wide and deep major groove (Figure 3), given the name "P-form helix" by the study authors [41]. Despite the much larger displacement of the bases from the helix axis, the base stacking in the P-form helix resembles that of an A-form DNA duplex. The sugars of a DNA strand adopt C3′-endo conformations with an average interphosphate distance of ≈6 Å, which is similar to A-type DNA and RNA, and allows the O1P oxygen from each DNA phosphate to form a hydrogen bond to the amide proton of each residue of the PNA backbone of the Hoogsteen strand [41]. More recent structural work by Rozners and co-workers confirmed that the PNA–dsRNA triplex had similar structural features [42]. The hydrogen bonding between PNA and RNA backbones is most likely the reason behind the >10-fold higher stability PNA–dsRNA triplexes [28–31] (compared to PNA–dsDNA) that favor structures having the ideal interphosphate distance of ≈6 Å. In contrast, the interphosphate distances in B-form structures (preferred by DNA) would be ≈7 Å. Most likely, PNA–dsDNA triplexes must pay an energy penalty by compromising between different stabilizing interactions that favor either B-like or A-like structures, which results in overall lower stability than the PNA-dsRNA triplexes where the stabilizing interactions are better aligned.

The crystal structure of a self-complementary PNA–PNA duplex was very similar to the P-form helix showing a wide helix (28 Å diameter) with a very large pitch of ≈18 base pairs per turn, compared to 10 and 11 base pairs per turn for DNA and RNA, respectively, and a nucleobase stacking pattern similar to that of the A-form RNA [43]. Another crystal structure of a partially self-complementary PNA–PNA duplex revealed PNA’s ability to combine the P-form Watson–Crick duplex with higher order structural features, such as reversed Hoogsteen base pairing, interstrand intercalation, triplex formation, and backbone chirality shifts [44]. A similar P-form helix having a wide and deep major groove and a shallow and narrow minor groove was also observed for an NMR solution structure of a self-complementary PNA–PNA duplex [45]. Taken together, these results confirmed that, while PNA was able to adopt to the conformations of DNA and RNA to some extent, the P-form was the naturally preferred helical conformation of PNA.

### PNA backbone modifications

PNA design was originally assisted by simple computer modeling that replaced the phosphodiester backbone of DNA with pseudopeptide linkages having the same number of atoms and linking bonds [2]. Not surprisingly, backbone modification has been a major focus of follow up attempts to improve the original PNA design. Early studies showed that maintaining proper distances (number of bonds) along the backbone and between the backbone and nucleobases of PNA was critical for effective nucleic acid binding as extension of either by additional methylene groups strongly decreased the binding affinity of PNA to either single- or double-stranded nucleic acids [46–48]. Furthermore, replacing amide linkages connecting the PNA’s backbone and the nucleobase with a tertiary amine also destabilized PNA complexes with complementary DNA [49]. The majority of the following studies focused on adding substituents to the original backbone for conformational control and improving PNA’s biophysical properties.

#### Conformationally constrained backbones

**Figure 4:**
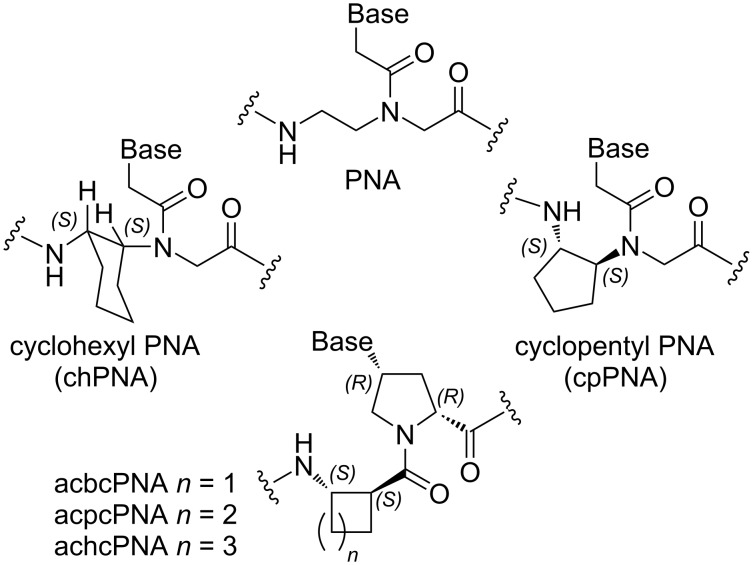
Structures of backbone-modified PNA.

Nielsen and co-workers [50] were the first to test restricting PNA backbone conformation by locking the backbone in a fused cyclohexane ring of either *S*,*S* or *R*,*R* configuration (chPNA, Figure 4). Both *S*,*S* or *R*,*R* chPNAs formed weaker complexes with complementary DNA and RNA than unmodified PNA [50]. Later, Kumar, Ganesh and co-workers [51–54] reported that either *S,R-* or *R,S*-modified chPNA had lower affinity for complementary DNA and RNA as well. The decreased binding affinity of chPNAs was most likely due to unfavorable dihedral angles for proper organization of PNA’s backbone. In contrast, Appella and co-workers found that restricting the backbone’s conformation with the fused *S,S*-cyclopentane ring increased the binding affinity of cpPNA (Figure 4) for complementary DNA and RNA compared to the unmodified PNA [55–56]. Govindaraju, Kumar and Ganesh [57–58] reported that isolated *S*,*R*- and *R*,*S*-cyclopentane modifications had variable effects on PNA binding affinity depending on their location (C-terminus, middle, or N-terminus) in PNA, while fully *S*,*R*- and *R*,*S*-modified cpPNAs were binding stronger to complementary DNA and RNA than the unmodified PNA. The *R*,*S*-modified cpPNAs appeared to be somewhat stronger binders than the *S*,*R*-modified counterparts [57–58]. Interestingly, PNAs having constrained backbones, including modifications that lowered affinity, were more sequence selective (less tolerant to mismatches) than unmodified PNA, which is important for development of diagnostics and therapeutics.

Recently, more detailed biophysical and structural studies on *S,S*-cpPNA by Appella and co-workers [59–60] show that the *S,S*-configuration of cyclopentane modification enforces dihedral angles of PNA backbone favorable for binding to complementary DNA. PNA binding affinity and sequence selectivity increase with increasing number of *S,S*-cyclopentane modifications allowing rational fine tuning of the complex stability. The recently published crystal structure of a duplex between completely modified *S,S*-cpPNA and a complementary DNA strand reveals preorganization of PNA backbone into a right handed-helix favorable for DNA binding [60]. At the time of this writing, binding of *S,S*-cpPNA to complementary RNA remains less well explored; however, other constrained backbone-modified PNAs reviewed above have shown stronger binding to RNA over DNA. *S,S*-cpPNA may be expected to follow this trend and, at this time, appears to be the most promising conformationally constrained PNA analogue.

Vilaivan and co-workers developed pyrrolidinyl PNA based on an α/β-dipeptide backbone that is one atom longer than the canonical PNA and contains two amide bonds and two cyclic moieties in one monomer (Figure 4) [61]. Cyclobutane-derived acbcPNA and cyclopentane-derived acpcPNA formed stable duplexes with matching DNA and RNA, while cyclohexane-derived achcPNA did not form complexes with either DNA or RNA, which was explained by unfavorable torsional angles and conformational rigidity of the cyclohexane backbone [62]. Interestingly and in contrast to other backbone-constrained PNAs, pyrrolidinyl α/β-dipeptide PNA formed PNA–DNA complexes having higher thermal stability compared to PNA–RNA complexes [63–64]. Most likely, the one atom longer PNA backbone, which is rigidified and preorganized by cyclic moieties, may align better with the B-form DNA helix rather than with the A-form RNA helix. While pyrrolidinyl α/β-dipeptide PNAs formed stable antiparallel duplexes with DNA and RNA with high mismatch intolerance, due to constrained nature, two pyrrolidinyl α/β-dipeptide PNAs had low ability to self-hybridize [62,65]. This property makes pyrrolidinyl α/β-dipeptide PNA especially suitable for double duplex invasion of dsDNA [66]. In general, pyrrolidinyl α/β-dipeptide PNA is another promising modification along with cyclopentane constrained PNAs studied by Kumar, Ganesh, and Appella.

#### PNA modified at alpha and gamma positions of the backbone

**Figure 5:**
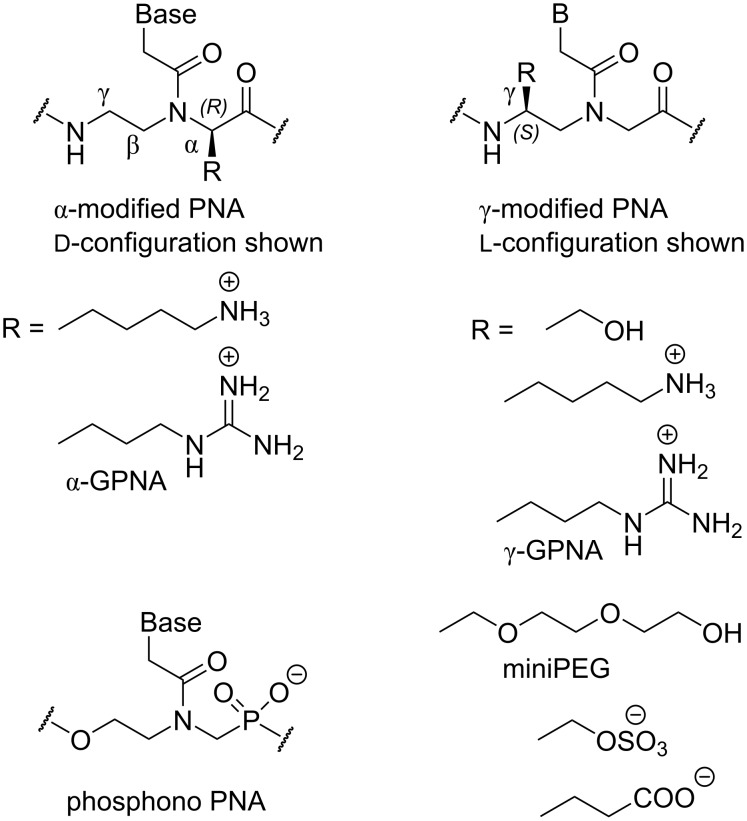
Structures of PNA having α- and γ-substituted backbones.

**α-Modified PNA:** Adding substituents to the *N*-(2-aminoethyl)glycine backbone has been an obvious starting point for PNA modification. Nielsen and co-workers were the first to replace the glycine residues in PNA backbone with various chiral amino acids [67–68]. Most of these α-modified PNA monomers (Figure 5) slightly reduced PNA binding affinity, with ᴅ-amino acids being somewhat better accommodated in the backbone than ʟ-amino acids and ᴅ-Lys being the only α-backbone modification that slightly increased PNA’s binding affinity to complementary DNA (but not RNA) [67]. Circular dichroism studies showed that the ᴅ-Lys modification induced a right-handed helical conformation favorable for DNA binding while the ʟ-Lys modification induced a left‐handed helical conformation that disfavored PNA binding to DNA [69]. Interestingly, a crystal structure of PNA having three α-ᴅ-Lys modifications in the middle [70] resembled the P-form helices formed by PNA–PNA and PNA–DNA–PNA more than the PNA–DNA structure [39].

Ly and co-workers synthesized α-modified PNAs derived from ʟ-arginine (α-GPNA, Figure 5) and showed that the positively charged guanidinium group increased the stability of PNA duplexes with complementary DNA and RNA, without compromising the sequence selectivity, and improved the cellular uptake of PNA [71]. The same group later demonstrated that GPNA derived from ᴅ-arginine formed more stable duplexes with RNA and was readily taken up by both human somatic and embryonic stem cells [72]. GPNA targeting the transcriptional start-site of the human E-cadherin gene had potent and sequence-specific antisense activity and was less toxic to the cells than the PNA–polyarginine conjugate [73]. Interestingly, the α-arginine modification in either ʟ- or ᴅ-configuration destabilized PNA–dsRNA triplexes [74].

**γ-Modified PNA:** Later studies focused on introducing substituents in the ethylenediamine moiety of the PNA backbone. Ly and co-workers showed that introduction of simple substituents, such as methyl (derived from ʟ-alanine) or hydroxymethyl (derived from ʟ-serine) at the γ-position (Figure 5) preorganized the PNA backbone in a right-handed helical structure favorable for stronger binding to complementary DNA and RNA [75]. The NMR structure showed that γ-methyl-PNA folded in a P-form helix similar to that observed for non-modified PNA but having more resemblance to A-form [76]. The γ-methyl-PNA helix was slightly more unwound and had a smaller twist angle than the P-helix of unmodified PNA. In a crystal structure, γ-methyl-PNA–DNA heteroduplex also adopted a P-form helix, with greater resemblance to A-form than B-form DNA, accommodating 15 base pairs per turn [77]. Dynamic transitions between different binding modes of γ-hydroxymethyl-modified triplex-forming PNAs have been also explored [78].

Englund and Appella showed that PNA containing γ-modifications derived from ʟ-lysine formed stronger duplexes with DNA and RNA, while γ-modifications derived from ᴅ-lysine decreased the stability of duplexes [79–80]. Ly and co-workers showed that γ-modified PNA derived from ʟ-arginine (γ-GPNA, Figure 5) were preorganized into a right-handed helix, which improved their binding to complementary DNA and RNA while retaining sequence selectivity [81]. As expected, the guanidine modifications greatly improved cellular uptake of γ-GPNA. Others have also investigated positively charged α- and γ-modifications of the PNA backbone, and most of them showed promising hybridization properties and improved cellular uptake [82–86]. Very recent work has used α- and γ-positions of the PNA backbone to attach additional nucleobases, which enable these “double face” PNAs to form higher order double and triple helical structures [87–88].

Ly and co-workers followed up on the promising conformational properties of γ-hydroxymethyl PNA by extending the side chain into a miniPEG modification (Figure 5). In addition to retaining the superior nucleic acid binding (due to preorganization of PNA’s backbone) miniPEG greatly improves aqueous solubility of PNA without causing any cytotoxicity [89]. Because of the superior binding properties, miniPEG-modified PNAs can invade any sequence of dsDNA using only Watson–Crick base pairing to recognize the target [89]. As will be discussed later in this review, PNAs having guanidine (γ-GPNA) and miniPEG γ-modifications are currently among the most promising PNA derivatives explored in medicinal chemistry and preclinical studies.

**Anionic PNA:** Anionic functionalities have been introduced in PNA to improve water solubility and better mimic DNA/RNA structure. One of the early studies was on chimeras of PNA and phosphono-PNA (Figure 5) that improved water solubility and in some cases resulted in stronger hybridization with complementary DNA and RNA [90]. The phosphono-PNAs retained the stability against nucleases. In another study, conjugation with glutamine phosphonate or lysine bis-phosphonate amino acid derivatives introduced up to twelve negative charges (phosphonate moieties) into PNAs [91]. The negative charges allowed cationic lipid-mediated delivery of PNAs to HeLa cells achieving sub-nanomolar antisense activity [91]. More recent studies introduced sulphate and carboxylate groups at the γ-position of PNA backbone (Figure 5) but neither modification showed promising hybridization profiles or improved cellular uptake [92–93].

#### Modified nucleobases in PNA

**Figure 6:**
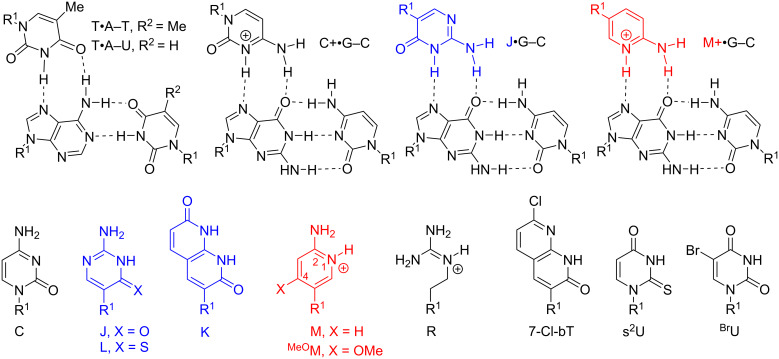
Structures of modified nucleobases in PNA to improve Hoogsteen hydrogen bonding to guanine and adenine. R^1^ denotes DNA, RNA, or PNA backbones.

**PNA nucleobases for Hoogsteen recognition of guanine:** As discussed in the Introduction, PNA was originally designed with the idea that the neutral backbone would improve binding properties of triplex-forming oligonucleotides. However, electrostatic repulsion is not the only weakness of triple helical recognition of nucleic acids. The binding affinity and sequence selectivity of triplex-forming oligonucleotides derives from thymine recognition of A–T (or A–U in RNA) base pairs (T•A–T or T•A–U triplet) and protonated cytosine recognition of G–C base pairs (C^+^•G–C triplet) via Hoogsteen hydrogen-bonding (Figure 6) [94]. A significant bottleneck for triple helix formation is the requirement for cytosine protonation to form the natural C^+^•G–C triplet. Because of the low p*K*_a_ of cytosine (≈4.5), formation of the C^+^•G–C triplet is unfavorable at physiological pH, which severely destabilizes the parallel triple helices and limits their applications in biological systems.

Two obvious strategies to solve this problem are to modify the cytosine heterocycle to either 1) increase the p*K*_a_ or 2) create neutral analogues of protonated cytosine. In the latter strategy, Ono et. al. introduced pseudoisocytosine (J, Figure 6) in triplex-forming oligonucleotides, alleviating the problem of unfavorable cytosine protonation [95–96]. Nielsen and co-workers replaced Cs with Js in the Hoogsteen strand of their original design of bis-PNAs in 1995 [34]. While J demonstrated weaker binding than C at pH 5, J enabled formation of relatively stable triplexes at physiological pH of 7.4. Later, the same research group reported that 1,8-naphthyridin-2,7-(1,8*H*)-dione (K, Figure 6), a bicyclic mimic of protonated cytosine, afforded stronger binding to G–C base pairs compared to J, most likely due to the increased surface area of the bicyclic nucleobases that enabled better π-stacking [97]. Despite the superior binding properties, the original report on K has not been followed up with more detailed studies and J remains the current gold standard for triple-helical recognition of G–C base pairs in PNA.

However, more recent studies show that J can be further optimized. Chen and co-workers reported that substitution of oxygen-4 of J with sulfur improved the Hoogsteen binding properties of 4-thiopseudoisocytosine (L, Figure 6) [98]. UV thermal melting and gel electrophoresis studies showed that L formed more stable L•G–C triplets than J when binding to dsRNA, which was suggested to be a combined effect of improved van der Waals contacts, base stacking, hydrogen bonding, and reduced dehydration energy [98]. Replacement of three Js with Ls increased the binding affinity of a PNA 8-mer ≈4-fold [98]. In addition, the sulfur modification removed the undesired ability of J to form a Watson–Crick base pair with G in single-stranded nucleic acids. This is important for avoiding off-target binding to single-stranded RNA and DNA in biological systems. L appears to be a promising improvement of J as a neutral analogue of protonated C for Hoogsteen recognition of G–C base pairs.

An alternative strategy that increases the basicity of cytosine through chemical modifications was pioneered by Povsic and Dervan who showed that addition of a 5-methyl substituent increased the stability of ^Me^C^+^•G–C triplet apparently through a subtle modulation of the p*K*_a_ and better π-stacking [99]. Several other research groups have further increased the p*K*_a_ value by removing electronegative substituents from C arriving at derivatives of 2-aminopyridine (M, Figure 6) as more basic nucleobases that improve binding of triplex-forming oligonucleotides at neutral pH [100–102]. Rozners and co-workers were the first to introduce M in triplex-forming PNAs targeting dsRNA [28]. Having a p*K*_a_ of ≈6.7, M is partially protonated at physiological pH 7.4, which facilitates fast binding and formation of strong triplex [28,30–31]. While all Hoogsteen triplets in Figure 6 are stabilized by two hydrogen bonds, because of the positive charge, M forms a significantly more stable M^+^•G–C triplet compared to either J•G–C or T•A–U in dsRNA [28,30]. In a recent study, replacement of six Js with Ms increased the binding affinity of a PNA 9-mer ≈100-fold [31]. Preliminary results suggest that PNA–dsDNA triplexes follow similar trends. Similar to L, M does not form a Watson–Crick base pair with G or any other natural nucleobase, which is important for avoiding off-target effects of triple-helical recognition in biological systems. M is unique among cationic RNA binding compounds, perhaps, because the protonation event is coupled with the Hoogsteen hydrogen bond formation. As a result, the partially protonated M strengthens the triple helix without compromising the sequence specificity of recognition [28,30–31].

As discussed above, guanidine groups have been attractive modifications because of their potential to improve cellular uptake of PNA. Interestingly, simple guanidine (R, Figure 6) as a single nucleobase replacement appeared to form a strong and selective R•G–C triplet; however, two consecutive R modifications destabilized the PNA–dsRNA triplex, most likely due to reduced ability of R to π-stack [103]. As expected, fluorescence microscopy showed improved cellular uptake of the cationic guanidinium-modified PNAs [103].

**Figure 7:**
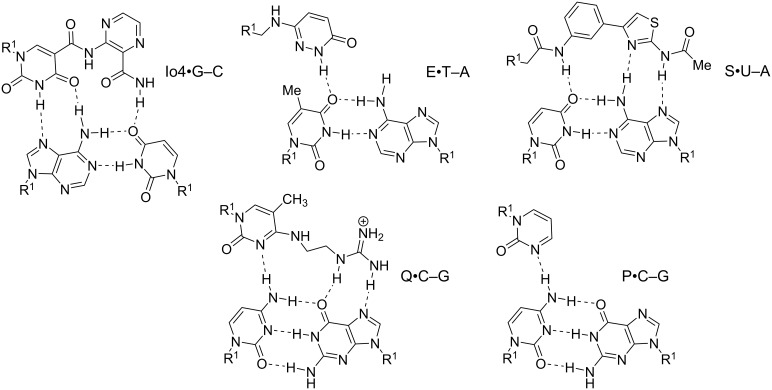
Proposed hydrogen bonding schemes for modified PNA nucleobases designed to recognize pyrimidines or the entire Hoogsteen face of the Watson–Crick base pairs. R^1^ denotes DNA, RNA, or PNA backbones.

**PNA nucleobases for Hoogsteen recognition of adenine:** Because the T•A–T triplets are reasonably stable under physiological conditions, development of novel nucleobases for Hoogsteen recognition of A has attracted less attention than the problem of C protonation discussed above. Similar to K, 7-Cl-bT, a substituted naphthyridine derivative (Figure 6), forms stronger Watson–Crick base pairs and Hoogsteen triplets with A, most likely because of enhanced stacking of the bicyclic π-system [104–105]. However, beyond the original studies, 7-Cl-bT has not been further explored for either duplex or triplex stabilization. Similar to L, substitution of thymine with 2-thiouracil (s^2^U) or 5-halouracils (e.g., ^Br^U, Figure 6) strengthens the Hoogsteen recognition of A. The stabilization provided by these nucleobases is most likely due to improved π-stacking, which may be sensitive to sequence context that needs to be further studied [106–107]. MacKay and co-workers designed an extended nucleobase based on isoorotic acid (Io4, Figure 7) to recognize the entire Hoogsteen face of the A–U base pair [108]. Io4 formed about a two-fold stronger triplet with the A–U base pair with good sequence selectivity. A PNA containing four consecutive Io4 nucleobases showed stronger binding to the complementary dsRNA than PNA containing four Ts suggesting that Io4 may be a promising alternative to T where stronger binding is desired [108].

**PNA nucleobases for Hoogsteen recognition of pyrimidines:** Triple helix formation, especially using tailored oligonucleotide analogues as PNA, could be a general and sequence specific approach for molecular recognition of dsDNA and dsRNA. However, the triple helical recognition has a severe sequence limitation – the requirement of polypurine tracts in target nucleic acids. Natural triple helices allow only T•A–T (or U•A–U) and C^+^•G–C triplets stabilized by two Hoogsteen hydrogen bonds (Figure 6) [94]. Analogous recognition of pyrimidines in hypothetical X•T–A or X•C–G triplets is complicated by two problems: 1) pyrimidines present only one hydrogen bond acceptor (C=O in T or U) or donor (-NH_2_ in C) in the major groove, and 2) the six-membered pyrimidine ring extends further out in the major groove than the five-membered ring of purines causing a steric clash with the incoming third nucleobase. In other words, the Hoogsteen face of Watson–Crick base pairs in the major groove is not isomorphous providing more space and better hydrogen bonding options for purines than for pyrimidines. Despite significant efforts by nucleic acid chemists, a universal solution to triple helical pyrimidine recognition is still missing [94,109].

Nielsen and co-workers introduced 3-oxo-2,3-dihydropyridazine (E, Figure 7), a synthetic nucleobase designed to form a single hydrogen bond with U in PNA–DNA–PNA clamps [110]. Their design connected E to the PNA backbone with a linker two atoms longer than in standard PNA, which was suggested to circumvent the 5-methyl group of thymine and enable hydrogen bonding to the 4-oxo group [110]. More recent work [111] has questioned the originally proposed hydrogen bonding scheme shown in Figure 7. In fact, all of the hydrogen bonding schemes in Figure 7, while reasonable, are proposed. They are not necessarily confirmed by structural studies. E was later used for recognition of U in PNA–dsRNA triple helices [112]. Most recent study from Sugimoto, Rozners, and co-workers showed that triplex formation by E- and M-modified PNAs was able to inhibit maturation of pri-microRNA hairpins in SH-SY5Y cells [113].

An alternative approach to pyrimidine recognition has been to develop extended nucleobases that bind the entire Hoogsteen face of a Watson–Crick base pair and take advantage of the hydrogen bonding options on the purine base as well. An extended nucleobase S (Figure 7) originally developed for triplex-forming oligonucleotides [114–115], was introduced in PNAs targeting U interruptions in polypurine tracts of dsRNA triplexes [111]. However, in PNA, S showed limited sequence specificity binding strongly to either U–A or C–G base pairs [111]. The low mismatch discrimination suggests that S may have binding modes other than the hydrogen bonding depicted in Figure 7, for example, intercalation as has been previously observed for other similar unnatural nucleobases in DNA [116]. At the time of writing, E remains the most commonly used PNA nucleobase for recognition of U–A base pairs in dsDNA and RNA [117].

Several heterocyclic nucleobases in triplex-forming oligonucleotides have been designed to form a single hydrogen bond with the exocyclic -NH_2_ of cytosine [94,109]. Rozners and co-workers [112] followed up on original work by Leumann [118] and showed that pyrimidin-2-one (P, Figure 7) could selectively recognize C–G, albeit with lower binding affinity than that of the standard Hoogsteen triplets. Despite the lower affinity, P-modified PNA formed a sequence specific triplex with a hairpin structure in the 5’-UTR of an mRNA, which inhibited ribosome assembly and suppressed mRNA translation in vitro and in cells [119]. This study was the first demonstration of the biological effect of binding of M- and P-modified PNAs to dsRNA in live cells. Recent work from our labs [120] systematically surveyed simple nitrogen heterocycles and found that the 3-pyridazinyl nucleobase formed significantly more stable triplets with C–G than other heterocycles, including P. Several groups have explored extended PNA nucleobases for recognition of C–G base pairs [121–122]. Chen and co-workers followed up on original work by Seidman [123] and showed that Q (Figure 7) in PNAs targeting dsRNA, recognized C–G base pairs with good selectivity. However, the stability of the Q•C–G triplet was reduced compared to T•A–U (≈8-fold) or L•G–C (≈24-fold) triplets [122]. Thus, an optimal solution for recognition of the C–G base pair in dsDNA and dsRNA remains elusive.

While the modified nucleobases reviewed above have given promising results, they typically lack either the binding affinity or selectivity of the natural triplets. This is especially true when the task is to recognize several pyrimidines, not just a single interruption of longer polypurine tract. Therefore, the search for new and better nucleobases for triple-helical recognition of any sequence of dsDNA or dsRNA remains an important goal and active area of research.

**Figure 8:**
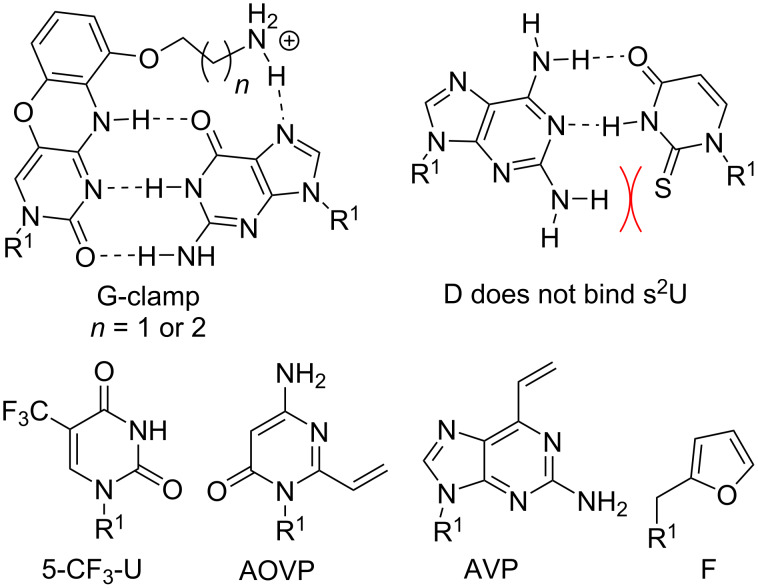
Modified nucleobases to modulate Watson–Crick base pairing and chemically reactive crosslinking PNA nucleobases. R^1^ denotes DNA, RNA, or PNA backbones.

**Nucleobases improving Watson–Crick recognition of PNA:** We previously noted that PNA forms duplexes with complementary DNA and RNA that are more stable than duplexes involving only natural nucleic acids. Nevertheless, nucleobase modifications can further improve the remarkable binding properties of PNAs. One of the most promising nucleobases for improving Watson–Crick binding is G-clamp (Figure 8), the phenoxazine-derived tricyclic analogue of cytosine [124]. The improvements in affinity provided by the G-clamp are likely a combined effect of superior π-stacking of the rigid and planar aromatic system, electrostatic attraction of the positively charged amine, and additional Hoogsteen hydrogen bonding [125]. Inserting just one G-clamp nucleobase into a PNA sequence increased the duplex melting temperature with complementary DNA or RNA by 13–20 °C while maintaining good mismatch discrimination [126].

Ganesh and co-workers found that substitution of the 5-position in uracil with fluorine or trifluoromethyl improved PNA binding affinity for complementary DNA and RNA [127]. Moreover, fluorination increased the cellular uptake of PNAs [127]. Fluorinated uracil derivatives are also useful probes for studying different binding modes of PNA using ^19^F NMR [128].

**PNA nucleobases for double duplex invasion:** Double duplex invasion (Figure 2F) critically depends on the ability of two PNAs to recognize each strand of dsDNA while not forming an unproductive PNA–PNA complex. Because the two DNA strands that are invaded are complementary, the two PNA strands have inherent complementarity as well. An elegant solution to this problem has been to use 2,6-diaminopurine (D) instead of adenosine and 2-thiouridine (s^2^U) instead of uridine as modified nucleobases in PNAs designed for double duplex invasion [129–130]. D and s^2^U form more stable Watson–Crick base pairs with T and A, respectively, than the natural A–T, but do not cross-bind in a D–s^2^U pair because of a steric clash between the 2-amino group of D and 2-thiocarbonyl group of s^2^U [129–130]. A recent report described an improved synthesis of s^2^U and s^2^T, which will help future applications of this currently somewhat underexplored technology [131].

**Chemically reactive crosslinking PNA nucleobases:** PNA has become a highly useful probe for detection of nucleic acids. Not surprisingly, chemists have developed reactive nucleobases for covalently crosslinking PNA and nucleic acid targets. 4-Amino-6-oxo-2-vinylpyrimidine (AOVP, Figure 8), a chemically reactive mimic of cytosine, exhibited selective crosslinking reactivity with thymine in DNA when incorporated at the terminal position of a PNA probe [132]. Interestingly, the activity of the crosslinking reaction was lower in RNA. Because AOVP functional groups do not match well any Watson–Crick base pairing scheme, AOVP lowered the stability of PNA duplexes with complementary DNA and RNA [132]. Similarly, vinyl-modified purine (AVP) effectively crosslinked with thymine in DNA and with uracil in RNA. The crosslinking resulted in inhibition of Dicer processing of microRNA precursors in vitro [133].

Furan (F, Figure 8) as a reactive nucleobase mimic was well accommodated in a duplex with DNA without decreasing its thermal stability [134]. Upon oxidation of the furan ring, F-modified PNAs reacted preferentially with cytosine and adenine and irreversibly crosslinked with ssDNA and dsDNA [134]. Covalent crosslinking of PNA with DNA or RNA upon hybridization is potentially highly useful for diagnostics and other applications as more stringent washing could be applied after hybridization with the complementary nucleic acid.

**Figure 9:**
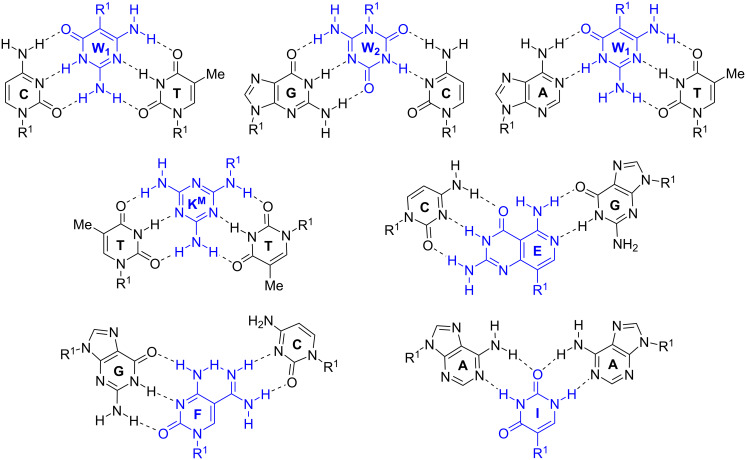
Examples of triplets formed by Janus-wedge PNA nucleobases (blue). R^1^ denotes DNA, RNA, or PNA backbones.

**Janus-wedge PNA triple helix:** McLaughlin and co-workers described a novel Janus-wedge triple helix (Figure 2G) where the wedge nucleobases (W_1_ and W_2_, Figure 9) of an incoming third PNA strand insert between two natural nucleobases hydrogen bonding with the Watson–Crick faces of the two DNA target strands from the major groove side [135–136]. This approach showed best results when invading DNA having consecutive C–T mismatches (C–W_1_–T triplet, Figure 9). W_2_ effectively bonded with the G–C base pair (G–W_2_–C triplet), but recognition of the A–T base pair (A–W_1_–T triplet) was significantly weaker and the Janus-wedge PNA was not able to invade a fully matched DNA duplex [136]. Bong and co-workers used melamine as a Janus-wedge nucleobase (K^M^, Figure 9) to organize two identical strands of oligothymidine DNA tracts (or oligouridine RNA tracts) on a peptide template to form peptide−DNA(RNA) triplex structures [137]. This approach was applied to induce RNA–RNA kissing loop dimerization and RNA–protein binding [138].

Ly and co-workers developed Janus-wedge nucleobases that invade both dsDNA and dsRNA Watson–Crick base pairs from the minor groove side. At the time of writing, three Janus nucleobases, E, F, and I (Figure 9) have been reported for recognition of C–G, G–C, and A–A base pairs, respectively [139–140]. While still in relatively early stages of development, the Janus-wedge triplex has already shown intriguing potential as a diagnostic or therapeutic approach for Huntington’s or related genetic diseases [139].

**Figure 10:**
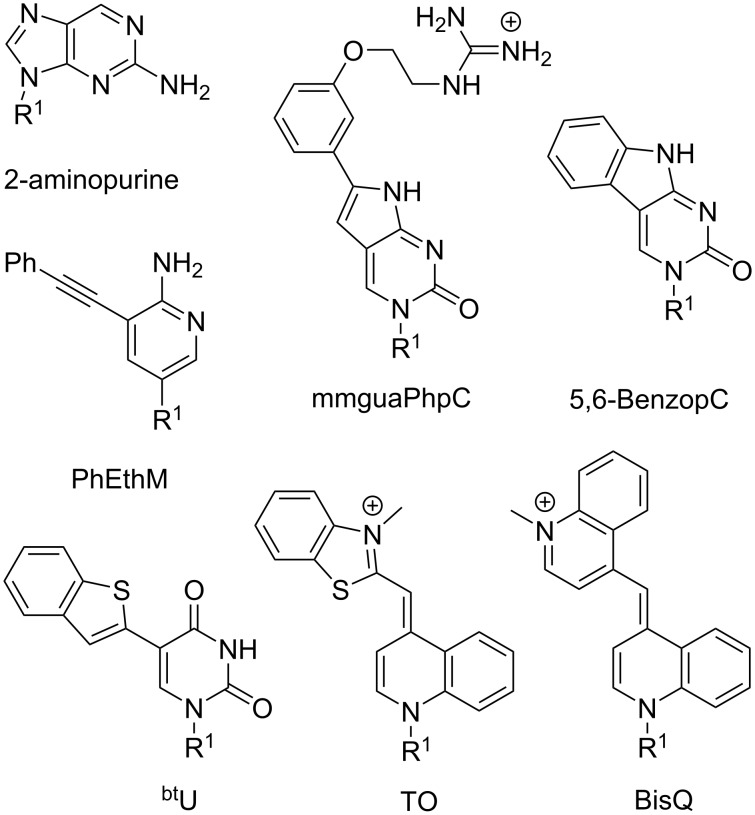
Examples of fluorescent PNA nucleobases. R^1^ denotes DNA, RNA, or PNA backbones.

**Fluorescent nucleobases in PNA:** Because PNA has become a key component of many assays and diagnostics, development of fluorescent nucleobases as labels for PNA has attracted considerable attention. 2-Aminopurine (Figure 10), a fluorescent structural isomer of adenine [141], was one of the first fluorescent nucleobases used in PNA [142]. Melting of a duplex formed by 2-aminopurine-modified PNA and complementary DNA increased the fluorescence signal, which had likely been quenched by adjacent nucleobases in the duplex [142]. Interestingly, quenching was also observed in a single stranded PNA alone, which diminished the applicability of 2-aminopurine in PNA probes. Hudson and co-workers developed several fluorescent PNA nucleobases derived from phenylpyrrolocytosine [143–145]. One of the most promising analogues, mmguaPhpC (Figure 10), formed a stronger base pair with G than the native C–G pair which was followed by a 30–70% decrease of emission intensity (dependent on the sequence context) upon hybridization with complementary DNA and RNA [145]. Another analogue, 5,6-BenzopC (Figure 10) had high quantum yield and superior base pairing properties, but its fluorescence was completely quenched upon hybridization with DNA and RNA [146]. Inspired by these findings, Cheruiyot and Rozners attempted to design fluorescent analogues of 2-aminopyridine; PhEthM (Figure 10) gave the best binding and fluorescence properties, but was strongly quenched upon formation of PNA–dsRNA triplex [147]. In general, quenching of PNA fluorescence upon binding to target DNA or RNA is less useful than the increase in signal intensity.

Chen and co-workers found that 5-benzothiopheneuracil (^bt^U, Figure 10) modified PNAs increased the fluorescence upon binding to dsRNA, acting as light-up triplex-forming PNA probes [148]. This was the first report of a modified natural nucleobase that did not quench the fluorescence upon hybridization [148].

Köhler and Seitz introduced thiazole orange (TO, Figure 10), an intercalator dye originally designed for DNA [149], as a forced intercalation (FIT) probe in PNA. Because of rotation around the methine bond connecting thiazole and quinoline, TO fluorescence is almost completely quenched in ssPNA, but increases significantly upon hybridization to the complementary DNA [150]. The intercalation of TO in PNA–DNA duplex restricts rotation around the methine bond enforcing planarity of the two TO’s aromatic system, which leads to fluorescence increase [151–152]. TO can be considered as a “universal base” due to its ability to pair equally well with each of the four natural DNA nucleobases [150]. Later, Nishizawa and co-workers used TO-modified triplex-forming PNAs as fluorescent probes sensitive to adjacent mismatched base pairs in dsRNA [153–154]. Replacement of thiazole in TO with another quinoline gives bis-quinoline (BisQ, Figure 10), a red-shifted PNA nucleobase analogous to TO [155]. Although binding of BisQ with all four natural DNA nucleobases has not been explored in detail, BisQ-modified FIT PNAs showed promising fluorescent enhancements and an ability to detect mismatches in live cells [155]. Overall, the TO- and BisQ-modified FIT PNAs are currently among the most promising fluorescent PNA probes.

While promising, the studies discussed in this section leave plenty of room for designing better fluorophores, especially, red-shifted dyes with stronger fluorescence enhancement. Future design of novel PNA nucleobases that enhance the fluorescence signal while selectively hybridizing to natural nucleobases will be highly beneficial for in vitro and in vivo probes and diagnostics.

#### Covalent PNA conjugates for delivery in cells and animal models

**Figure 11:**
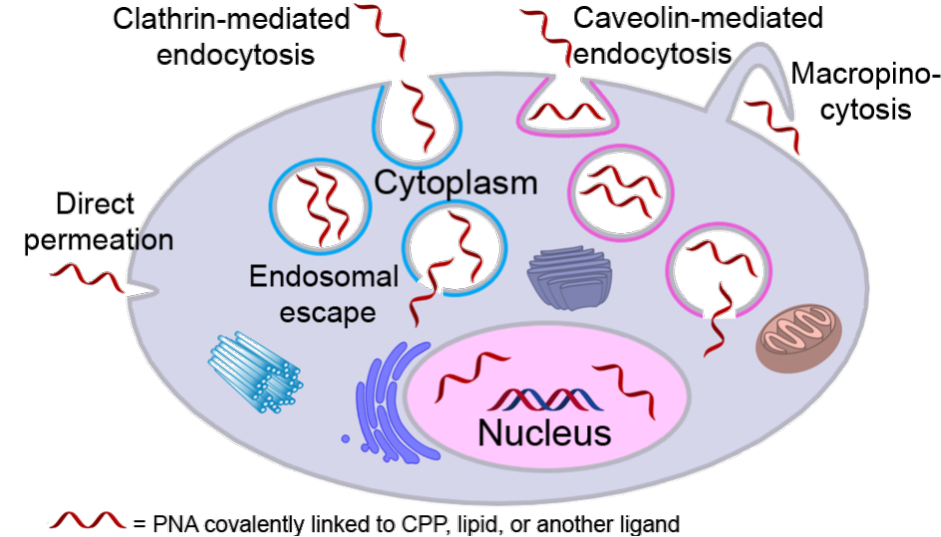
Endosomal entrapment and escape pathways of PNA and PNA conjugates.

Delivery and uptake of oligonucleotides to target tissues and cells is one of the greatest challenges for development of nucleic acid detection probes and therapeutics [14]. This problem is especially critical for in vivo applications of PNA because unmodified PNA, despite being charge neutral, does not readily cross cellular membranes [16,156–158]. Not surprisingly, the first demonstration of PNA-mediated suppression of gene expression by Babiss and co-workers used nuclear micro injection [38]. Another common method for PNA delivery has been electroporation [119,159–160]. Looking forward, conjugation of PNA with various delivery enhancing compounds, most notably cell-penetrating peptides (CPP) that deliver the conjugates mainly through endocytosis (Figure 11) has become one of the most promising approaches to improving cellular uptake of PNA [161–162]. However, the uptake of most PNA–CPP conjugates is limited by endosomal entrapment. While the uptake can be improved either by increasing the concentration of PNA–CPP conjugates or by using endosomolytic compounds (for example, chloroquine or calcium ions) this leads to toxicity that is not viable for in vivo applications [163]. Inefficient and incomplete release from endosomes remains an unsolved problem for PNA–CPP conjugates [164]. In this section we review the initial approaches and some of the most promising and foundational studies undertaken in addressing the cellular delivery issue using the covalent conjugation of PNA to delivery enhancing compounds.

**Figure 12:**
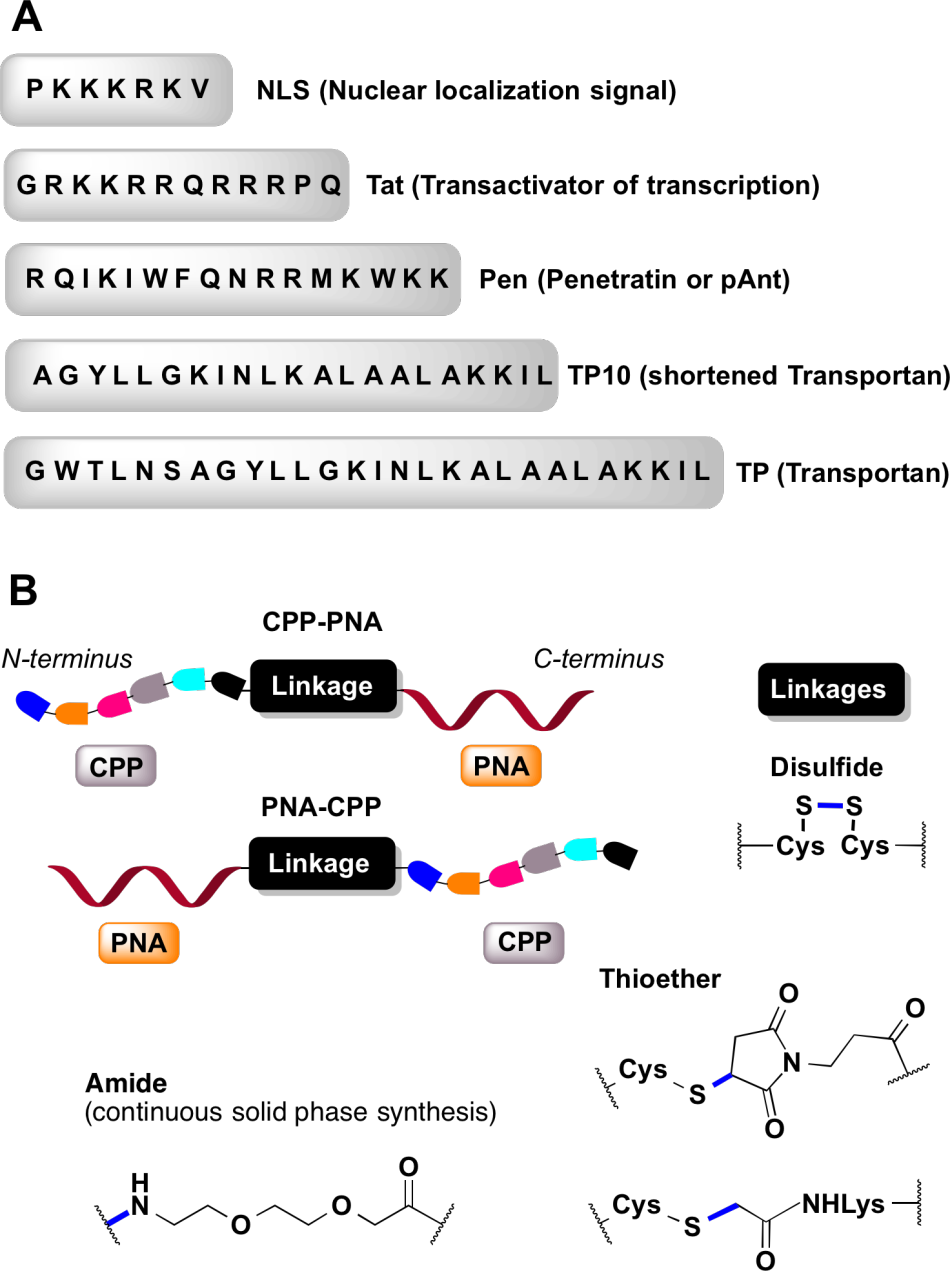
(A) representative cell-penetrating peptides (CPPs), (B) conjugation designs and linker chemistries.

**Cell-penetrating peptides derived from natural proteins:** The initial success of PNA delivery involved PNA conjugates taken up by receptor-mediated endocytosis. Pardridge and co-workers successfully demonstrated in vivo delivery and blood-brain barrier crossing of PNAs by intravenous administration of PNA conjugated to OX26 murine monoclonal antibody to the rat transferrin receptor [165]. The limitation of this strategy was complexity of the construct and lack of clear evidence for the cellular uptake. The first report of using the PNA–peptide conjugate approach involved the conjugation of PNA to (ᴅ)-insulin-like growth factor 1 peptide (IGF1) that enabled the delivery to cells expressing the IGF1 receptor [166]. Later developments adopted CPPs derived from natural proteins (Figure 12A), such as penetratin (16-amino acid peptide from the third helix of the Antennapedia homeodomain) [167], Tat (14-amino acid peptide from HIV-1 TAT protein) [168], and transportan (chimeric 27-amino acid peptide derived from galanin and mastoparan) [169].

Corey and co-workers were the first to demonstrate that conjugation of an 11-mer PNA to penetratin peptide enabled uptake of the conjugate in DU145 cancer cells as analyzed by fluorescence-activated cell sorting (FACS). However, the conjugate did not inhibit the targeted human telomerase in cells [170]. Langel and co-workers conjugated antisense PNA targeting mRNA of galanin receptor type 1 (GalR1) through disulfide linkages to transportan and penetratin peptides. The PNA–peptide conjugates were effectively internalized in human Bowes melanoma cells and in vivo in rats [171]. Transportan peptide localized the PNA in membranous structures of cells, while the penetratin conjugate preferred nuclear localization. The conjugates inhibited ^125^I-galanin binding in Bowes cells with 91% efficiency of PNA–penetratin (3 μM) and 83% of PNA–transportan (1.5 μM), which compared favorably with 5% efficiency of antisense DNA (10 μM) and 37% of phosphorothioate-modified antisense DNA (12 μM). In rats, intrathecally administered PNA–penetratin conjugate (3 × 10 μL of 150 μM) caused a 40% decrease in ^125^I-galanin binding in spinal cord sections compared to rats treated with the saline control. The PNA–peptide conjugates showed no toxicity in these studies [171].

Boffa and co-workers conjugated antigene PNA to a nuclear localization signal (NLS) peptide (PKKKRKV, Figure 12A), and showed that the PNA–NLS conjugates localized predominantly in the nucleus rather than in the cytoplasm of Burkitt's lymphoma cell lines (BRG, BJAB, HBL2) [172]. The opposite trend was observed for unmodified PNA or PNA conjugated to a scrambled-NLS peptide (KKVKPKR). UV melting studies showed that the conjugation of basic NLS peptide to PNA did not influence the binding ability for the complementary DNA. In BRG cells at 10 μM concentration, PNA–NLS targeting *c-myc* gene reduced its expression by 75% compared to controls having scrambled PNA or peptide sequence, or unmodified PNA [172].

Peschke and co-workers conjugated a dual peptide construct built of penetratin (for cytosolic delivery) and NLS (for nuclear delivery) at the N-terminus of PNA and demonstrated efficient delivery and distribution of the conjugate (100 nM) in the nucleus of DU 145 or R3327-AT1 prostate tumor cells [173]. Importantly, the efficient delivery of PNA to the nucleus was achieved only when the penetratin and NLS peptides were connected by a cleavable disulfide linkage (Figure 12B). PNA conjugates with penetratin only or dual peptide with a non-cleavable linker localized mostly in the cytosol with very little nuclear delivery. Confocal imaging studies of a fluorescently labeled dual peptide–PNA conjugate revealed initial cytosolic delivery, followed by cleavage of the disulfide linkage in cytosol and nuclear uptake of NLS–PNA. The ability to achieve delivery and diffused nuclear localization of PNA using only 100 nM concentration of the dual peptide conjugate was a significant achievement; however, this study did not demonstrate antisense or other biological effects of the PNA–penetratin conjugate [173].

Nielsen and co-workers compared the cellular uptake of unmodified PNA with α-backbone-modified PNA derived from lysine (T_Lys_-PNA, Figure 5), CPP (Tat or Penetratin, Figure 12A) alone, and PNA–CPP conjugates in HeLa (cervical carcinoma), SK-BR-3 (breast carcinoma) and IMR-90 (fetal lung fibroblast) monolayer cells, as well as in H9 (lymphoid) and U937 (monocytic) suspension cells [174]. At 2.0 μM concentration, T_Lys_–PNA and PNA–CPP were readily taken up by the three monolayer cell lines but were confined exclusively to the cytosolic vesicular compartments. T_Lys_–PNA and PNA–CPP showed very weak membrane staining in H9 cells and no uptake in U937 cells. The vesicular uptake was time, temperature and concentration dependent indicating an endocytic pathway (Figure 11). PNA alone and CPPs alone were not taken up in cells under the experimental conditions used in this study. It was also noted that depending on the cell type, the PNA–CPP conjugates were cytotoxic above 5–10 μM [174].

**Figure 13:**
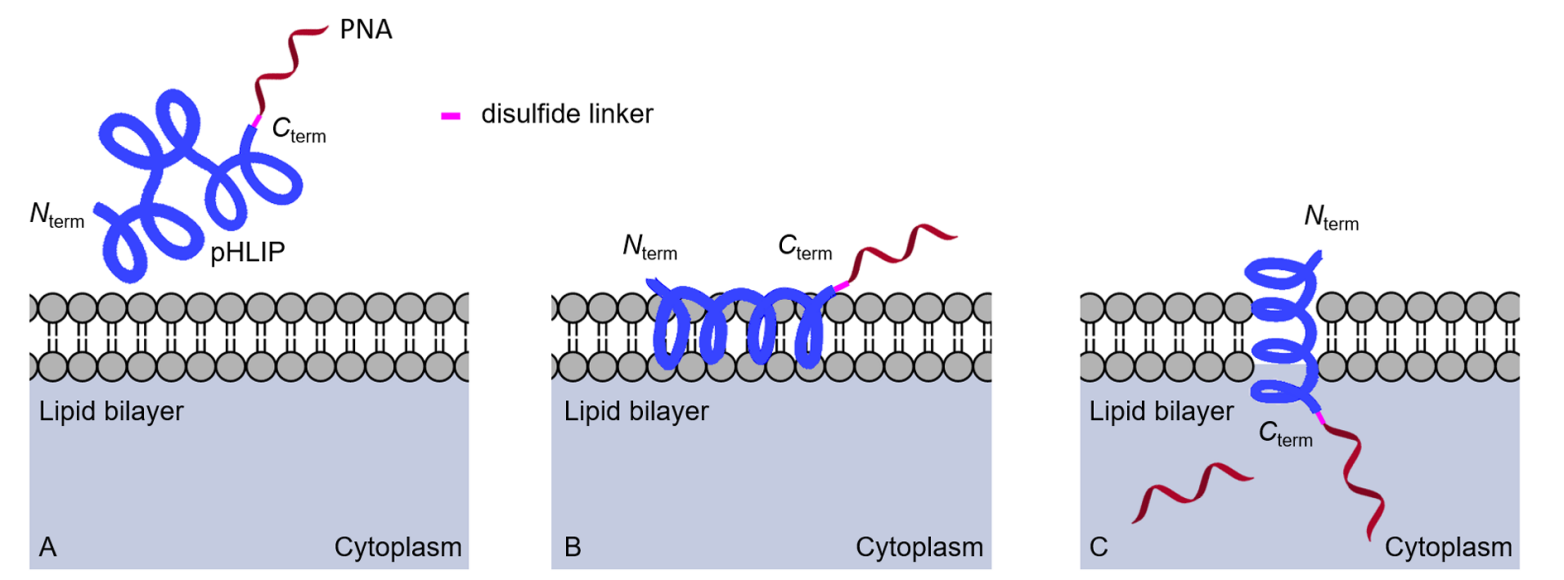
Proposed delivery mode by pHLIP-PNA conjugates (A) the transmembrane section of pHLIP interacting with lipid bilayer, (B) low surface pH leads to partial protonation of negative residues triggering interfacial helix formation and deeper partitioning into lipid bilayer, and (C) the transmembrane helix formation and release of PNA into cytosol by disulfide cleavage.

Gait and co-workers studied the effect of different CPPs and linkers (Figure 13) on activity of PNA conjugates targeting the apical stem-loop of TAR at the 5′-end of HIV-1 RNA [175]. In this study, the inhibition of HIV-1 Tat-mediated *trans*-activation in HeLa cells was monitored using an integrated double-luciferase reporter system [175]. PNAs conjugated through a stable amide linker to various CPPs (Figure 12B) showed no inhibitory activity at 2.5 µM while cell viability remained >95%. Co-administration with 100 μM chloroquine showed significant to weak inhibitory activity for Tat–PNA, TP–PNA, TP10–PNA, NLS–PNA–Tat, PNA–TP10, and Tat–PNA–NLS (Figure 12). However, no inhibition activity was recovered for NLS–PNA, PNA–NLS, and K_8_–PNA–K. Some conjugates having cleavable linkers, such as, Tat–S–S–PNA, Pen–S–S–PNA, and R_9_F_2_–S–S–PNA showed no inhibitory activity at 2.5 μM either with or without 100 μM of chloroquine. Three conjugates having cleavable linkers, R_6_-penetratin–S–S–PNA, TP–S–S–PNA and TP(int)–S–S–PNA showed significant levels of inhibitory activity at 2.5 μM, which was further increased in the presence of 100 μM chloroquine, while maintaining sequence-specificity. Overall, the poor activity of most of the CPP–PNA conjugates in the nucleus was attributed to the poor escape from endosomes or other membrane-bound compartments [175].

Cao and co-workers conjugated a PNA targeting the direct repeats of hepatitis B virus (HBV) to Tat peptide using 1,4-addition of C-terminal cysteine thiol on Tat to N-terminal maleimide on PNA [176]. The resulting Tat-PNA conjugate showed excellent in vitro and in vivo antiviral properties. In HepG2.2.15 cells, the Tat–PNA conjugate blocked expression of HBV DNA, RNA and proteins (HBeAg, HBsAg, HBV core, x protein, reverse transcriptase) indicating multiple modes of action, in contrast to the single mode of reverse transcriptase inhibition by the clinically approved drug lamivudine. The Tat–PNA conjugate was not toxic at 100 μM in multiple cell lines from hepatocytes and erythrocytes. Intravenous injection of the Tat–PNA conjugate at 50 mg/kg in mice did not cause acute toxicity or immune response as judged by levels of IgG and IgM measured by ELISA. The Tat–PNA conjugate suppressed HBV DNA concentration in serum of mice infected with HBV as measured by quantitative real time PCR (qRT-PCR) to 1.4 × 10^4^ copies/mL, which compared favorably with 1.2 × 10^4^ copies/mL in lamivudine treated mice and was lower than 6.9 × 10^4^ copies/mL in untreated mice. In mouse liver tissues, HBV core-protein-positive hepatocytes were reduced to 1.7% compared to 4.5% in untreated mice. In addition, very low levels of viral antigens (HBeAg and HBsAg) were observed in the blood of mice treated with the Tat–PNA conjugate [176]. These results suggested that targeting of direct repeats of HBV using PNA–CPP conjugates might be explored as a potential therapeutic strategy against HBV.

Engelman and co-workers discovered that a 36-residue polypeptide derived from transmembrane helix C of bacteriorhodopsin spontaneously inserts into the lipid bilayer under slightly acidic conditions [177]. Follow-up studies developed a pH-low insertion peptide (pHLIP) that translocates impermeable drug molecules specifically across the membranes of cells with low surface pH ≈ 6 (Figure 13) [178–180]. Peptides of the pHLIP family typically contain a transmembrane peptide sequence, which is essential for interactions with the lipid bilayer of cells, and short flanking sequences at the C- and N-terminus that promote membrane insertion and peptide solubility [178,180].

Slack and co-workers conjugated a 23-mer PNA targeting miRNA-155 to the C-terminus of pHLIP through a cleavable disulfide linkage. In A549 and DLBCL tumor cell lines, enhanced delivery of pHLIP–PNA was observed at the slightly acidic extracellular pH of tumor cells [181]. Intravenous administration of the pHLIP-PNA conjugate (2 mg/kg) in two mouse models, *mir-155^LSLtTA^* subcutaneous flank model and *mir-155^LSLtTA^* model of lymphoma was studied [181]. The systemically administered pHLIP-PNA accumulated in the enlarged lymph nodes of transgenic *mir-155^LSLtTA^* mice. Significant reduction in the tumor growth was achieved in the flank tumor model. The survival time of 11 days for pHLIP–PNA treated mice compared favorably with 7 days for mice treated with commercial locked nucleic acid (LNA) anti-miR at 17–40-fold higher concentrations than pHLIP–PNA. The pHLIP–PNA conjugate not only delayed the tumor growth but also suppressed the metastatic spread of neoplastic lymphocytes to other organs with no clinical signs of distress, toxicity or renal damage [181].

Glazer and co-workers conjugated pHLIP via a disulfide linkage to antisense γ-miniPEG-modified PNA (Figure 5) targeting nonenzymatic-NHEJ factor Ku80 mRNA [182]. In human lung adenocarcinoma (A549) cells at pH 6.2, this pHLIP–PNA conjugate showed ≈45% reduction of Ku80; no activity observed at pH 7.8. Systemic delivery of the pHLIP–PNA conjugate (5 mg/kg) in mice bearing DLD1-BRCA2KO human colon cancer xenograft reduced the Ku80 expression by ≈40%. Similar partial suppression was observed in EMT6 tumors as well. No significant toxicity or immune response was noted in mice treated with the pHLIP–PNA conjugate and, unlike with many anticancer therapeutics, no bone marrow toxicity was observed [182].

Pentelute and co-workers achieved efficient cytosolic delivery of PNA using the two nontoxic components of the anthrax toxin, the protective antigen (PA) and the N-terminal domain of lethal factor (LF_N_) [183]. The antisense PNA was conjugated to the C-terminus of LF_N_ through sortase-mediated ligation. The advantage of LF_N_/PA mediated delivery was demonstrated by the 100- to 1000-fold higher antisense activity at nanomolar concentrations (250 nM LF_N_–PNA and 50 nM PA protein) in cancer cell lines compared to PNA alone or Tat–PNA conjugates (no activity up to 5 μM). The robustness of LF_N_/PA delivery system was demonstrated by delivering PNAs across a panel of nine cancer cell lines from breast and blood lineages. The PNA–LF_N_ conjugate (100 nM) in the presence of PA protein (50 nM) caused a significant decrease in the viability of BT549 and HCC1954 breast cancer cells (50%) and Toledo and HUT 78 blood cancer cells (80%). Neither the length nor the sequence of PNA affected the translocation efficiency using the LF_N_/PA delivery system; however, neutralizing antibodies produced by the immune system remained a critical challenge for this delivery system [183].

**Synthetic cell-penetrating peptides:** Kole and co-workers compared PNAs conjugated to one, two, and four lysines (PNA–K, PNA–K_2_, and PNA–K_4_) with negatively charged 2′-*O*-alkyl oligonucleotide derivatives and neutral morpholino phosphorodiamidates (PMOs) in HeLa cells [184]. Passive uptake studies by FACS showed that PNA–K, PNA–K_2_, PNA–K_4_, and PMOs crossed the cellular membrane and gained access to the nucleus more readily than the anionic oligonucleotide analogues. In a splicing correction assay, increasing the number of lysines in the series PNA–K, PNA–K_2_, and PNA–K_4_ correlated with increased splicing modulation activity with EC_50_ of 4.7, 3.3, and 2.1 μM, respectively. The uptake mechanism was similar to that of PNA–penetratin conjugates. MTT assay showed no toxicity associated with PNA–K_4_ even at 10 μM. In the clinically relevant β-thalassemia model, in the absence of transfection reagents, the correct splicing of IVS2-654 human β-globin pre-mRNA was four-fold higher with PNA–K_4_ compared to PMO as measured by qRT-PCR [184].

Kole and co-workers also compared antisense activity of PNA–K_4_, PMO and 2′-O-methoxyethyl phosphorothioate (2′-O-MOE-PS) oligonucleotides in EGFP-654 transgenic mice [185]. In this model, antisense activity restores correct splicing and expression of enhanced green fluorescence protein (EGFP) providing an easy readout of in vivo activity. Systemically injected 2′-O-MOE-PS and PNA–K_4_ oligomers showed sequence-specific antisense activity in cardiac muscle, cortex of kidney, liver hepatocytes, lung and small intestine, while PMOs had weak or moderate activity in all these tissues and PNA–K was completely inactive. PNA–K_4_ was the most effective antisense in all the tissues except small intestine where 2′-O-MOE-PS was more effective [185]. No antisense activity was observed in brain, skin and stomach with any of the oligomers.

Follow up studies by Corey [186–187] and Gait [164,175,188] and co-workers demonstrated that PNAs conjugated to short oligolysine peptides (four to eight residues) were efficiently taken up in cancer cell lines. Later studies demonstrated delivery and antisense activity of PNA–K_8_ and K–PNA–K_3_ conjugates in mice [189–190]. The cellular uptake of these simple conjugates was further optimized by addition of a terminal thiol group (cysteine in C–K–PNA–K_3_) [191].

Corey and co-workers compared PNA–(AAKK)_4_, PNA–NLS, and unmodified PNA delivered by complementary DNA/lipid co-transfectant [192]. They found that PNA–(AAKK)_4_ and PNA–NLS were taken up in cultured cells but required higher PNA concentration to achieve the same uptake as that of DNA/lipid-mediated PNA delivery. In the absence of DNA/lipid co-transfectant, unmodified PNA and NLS–PNA did not inhibit expression of the human caveolin 1 (hCav-1) gene, while PNA–(AAKK)_4_ reduced the expression of hCav-1 with IC_50_ 2 μM.

Wright and co-workers enhanced the antisense activity of the PNA–K_8_ conjugate in the presence of PA protein (the protective antigen from anthrax) in CHO and HeLa cells [193]. Interestingly, reducing the lysine tail at the C-terminus to four in PNA–K_4_ reduced the antisense activity ≈2-fold. Reducing the lysine tail further from four to two residues completely eliminated the antisense activity, highlighting the importance of lysine conjugation at the C-terminus of PNA. Administration of PNA–K_8_ (300 nM) and PA protein (2 × 300 ng/mL) corrected the β-globin splice defect in cultured erythroid precursor cells from a patient with β-thalassemia, while no correction was observed with PNA–K_8_ alone, highlighting the role of PA protein in delivering the PNA into cells [193].

Nielsen and co-workers demonstrated the antibacterial properties of PNAs by targeting 23S rRNA using unmodified bis-PNA, which inhibited the growth of the AS19 strain of *E. coli* that had a compromised and permeable cell membrane [194]. However, no growth inhibition was observed in case of the membrane intact K12 strain of *E. coli* [194]. In a later study by Good and Nielsen, conjugation of an antisense PNA targeting the *lacZ* gene in *E. coli* to a synthetic antibacterial peptide (KFF)_3_K [195] composed of cationic lysine and hydrophobic phenylalanine, inhibited growth of *E. coli* K12, with a minimal inhibitory concentration of 3.0 μM, while free peptide and unmodified PNAs showed no activity [196]. A (KFF)_3_K–bis-PNA conjugate targeting mRNA of acyl carrier protein (*acp*P) at 2.0 μM concentration reduced the colony forming units (CFU) from 10^5^ per mL to zero in three hours. Most importantly, the (KFF)_3_K–bis-PNA conjugate at 2.0 μM fully cured the *E. coli* infection in *E. coli* K12 infected HeLa cells without harming the host HeLa cells [196].

**Figure 14:**
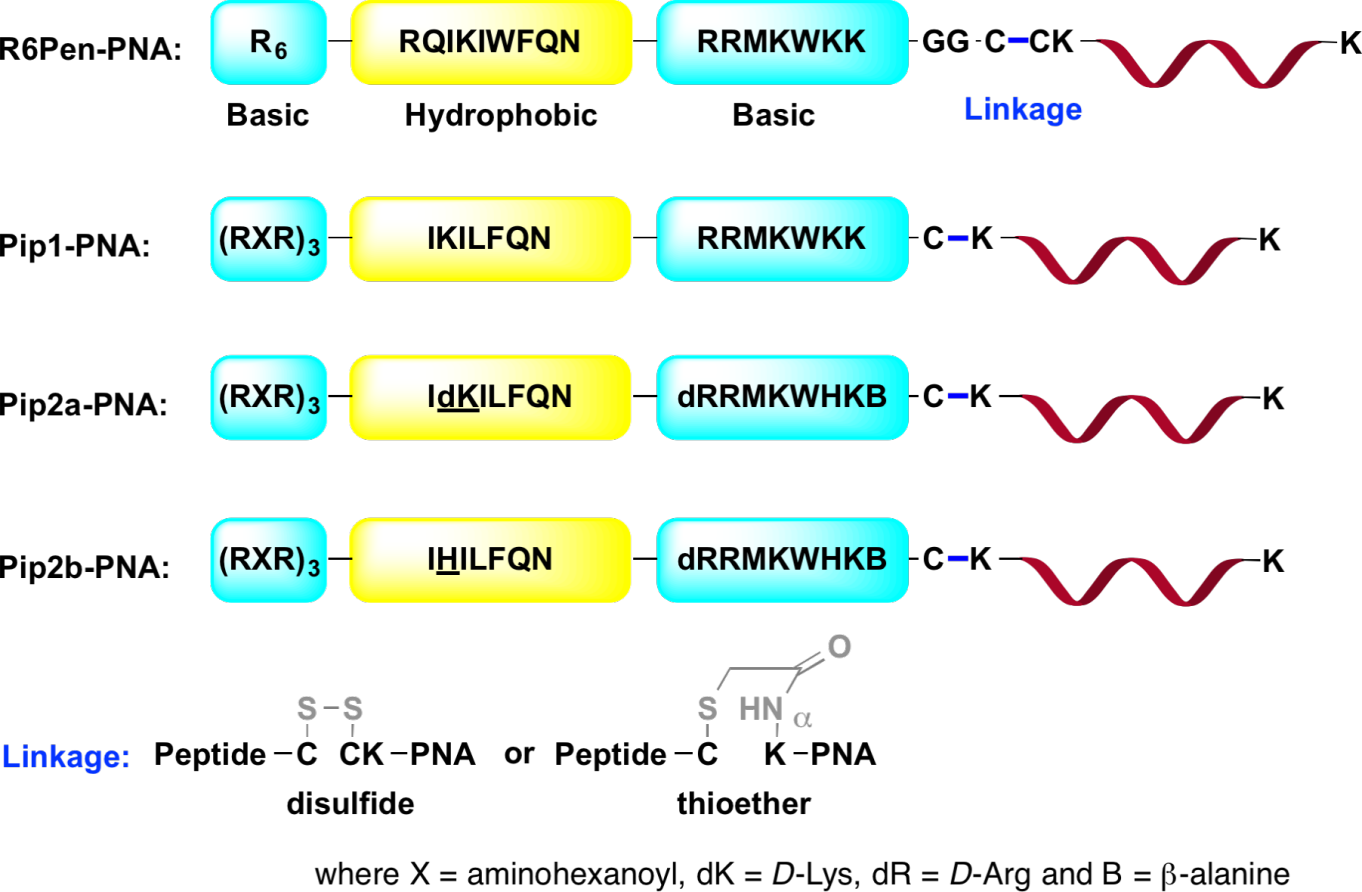
Structures of modified penetratin CPP conjugates with PNA linked through either disulfide (for study in HeLa pLuc705 cells) or thioether bonds (for study in cultured *mdx* mouse myotubes or mouse model).

Gait and co-workers developed a series of CPPs called PNA internalization peptides (Pip, Figure 14) by combining and optimizing the amino acid sequences of (RXR)_4_, previously developed for delivery of charge-neutral PMOs [197], and penetratin CPPs [198]. The uptake of Pip–PNA conjugates followed the pathway of clathrin-dependent endocytosis, as previously established for Tat–PNA and (RXR)_4_–PMO conjugates [199]. In HeLa pLuc705 cells, the Pip1–PNA conjugate showed higher splice correction activity (EC_50_ = 0.5 μM) than R_6_Pen–PNA (EC_50_ = 1.0 μM) or (RXR)_4_–PNA (EC_50_ = 3–4 μM) conjugates, but was fully cleaved within 1 hour in 20% mouse serum. Pip1 was further optimized into two serum-stabilized peptides, Pip2a and Pip2b (both differ by a single amino acid at position 11, underlined in Figure 14). In cultured *mdx* mouse myotubes, Pip2a–PNA and Pip2b–PNA conjugates targeting the exon 23 mutation in the dystrophin gene induced significant exon skipping at 1 and 2 μM, while maintaining the cell viability above 80% at concentrations up to 5 μM. The Pip1–PNA and (RXR)_4_–PNA conjugates induced a small amount of exon skipping at 2 μM. In a mouse model of Duchenne muscular dystrophy (DMD), a single dose of 5 μg of Pip2a–PNA and Pip2b–PNA conjugates showed a significant increase in the dystrophin-positive myofibers [198].

Gambari and co-workers conjugated PNA with the well-established octaarginine CPP [200] and used R_8_–PNA in Glioma cells to inhibit microRNA-221 (miRNA-221), which down regulates the expression of p27^Kip1^ among several other genes [201]. Surface plasmon resonance confirmed that conjugation with the highly cationic R_8_ peptide did not compromise sequence specificity of the R_8_–PNA conjugate. FACS and confocal microscopy showed high levels of uptake of the R_8_–PNA conjugate at 2.0 μM compared to unmodified PNA in U251, U373, and T98G Glioma cells. Strong miRNA-221 inhibitory effects were observed at 2 μM with the R_8_–PNA conjugate while no inhibitory effects were observed with an unmodified PNA or R_8_–PNA conjugate having mutated PNA. Moreover, the R_8_–PNA conjugate did not inhibit the closely related miRNA-210 and -222, members of the same family as miRNA-221 [201].

**Figure 15:**
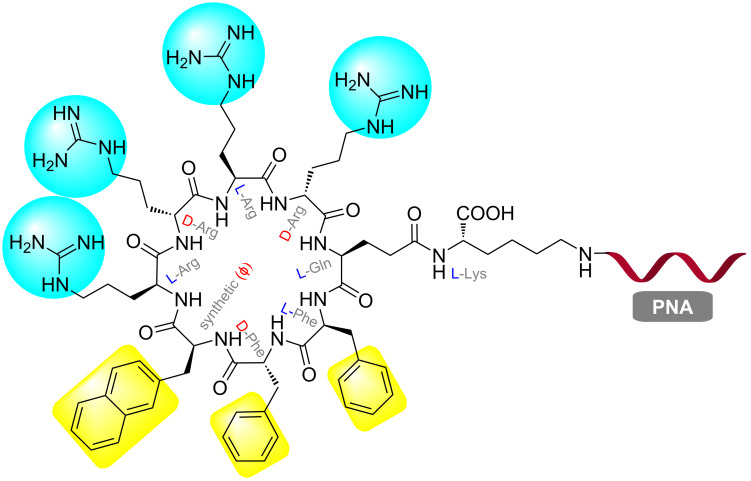
Chemical structure of C_9_–PNA, a stable amphipathic (cyclic-peptide)–PNA conjugate.

Searching for a general membrane transporter for therapeutic agents, Pei and co-workers discovered that cyclic peptides were ≈20-fold more efficient for cytosolic delivery in HeLa cells compared to common CPPs, such as, Tat and R_9_ [202]. Yavin and co-workers adopted this strategy and synthesized a PNA conjugate with a cyclic peptide C_9_–PNA (Figure 15) [203]. After incubation at 500 nM for 3 h, C_9_–PNA showed significant uptake in U87MG cells as judged by live cell fluorescence microscopy and FACS analysis, compared to less efficient uptake of K_4_–PNA under the same conditions. In U87MG cells, which are difficult to transfect, at 500 nM concentration C_9_–PNA and K_4_–PNA reduced the miRNA-155 levels by ≈80 and 65%, respectively [203].

**Figure 16:**
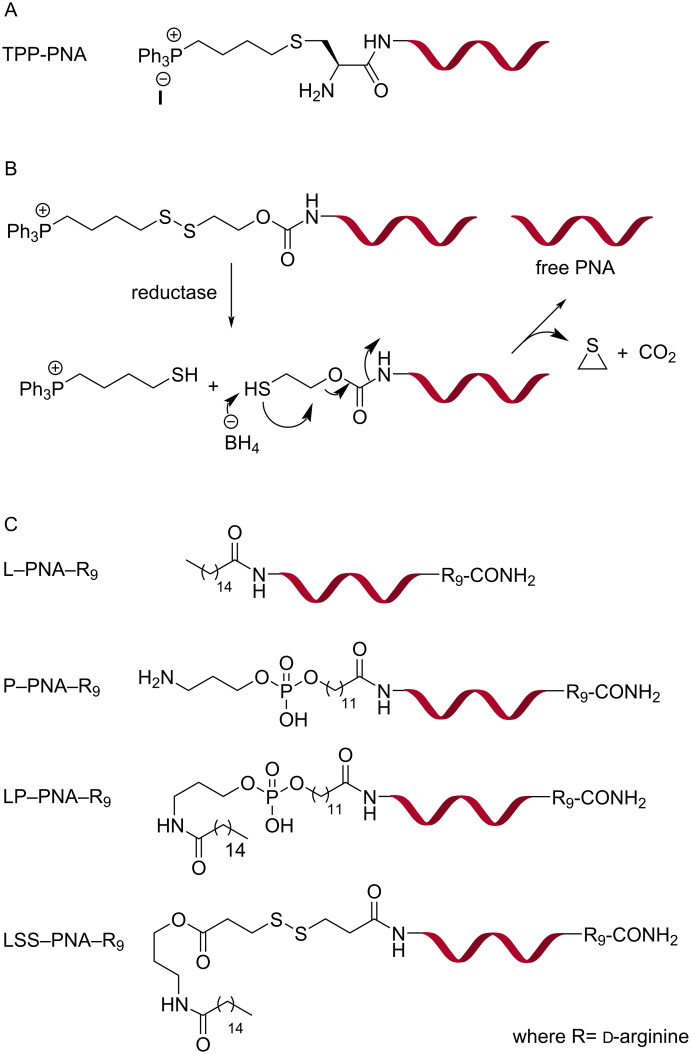
Structures of PNA conjugates with a lipophilic triphenylphosphonium cation (TPP–PNA) through (A) thioether and (B) cleavable disulfide linkage; (C) PNA–R_9_ conjugates with lipids, phospholipids and cleavable lipids.

**Lipid-based delivery of PNA:** Murphy and co-workers conjugated a lipophilic phosphonium cation (TPP, Figure 16A) through a thioether linkage to a PNA targeting a point mutation in mitochondrial DNA [204]. The TPP–PNA conjugates (1.0 μM) were efficiently taken up in mitochondria of cultured human cells, myoblasts and fibroblasts, driven by the inner membrane potential across the lipid bilayer of mitochondria as evidenced by microscopic images. Mitochondrial localization of the TPP–PNA conjugate was noticed after 4 h but, surprisingly, did not show inhibition of target mitochondrial DNA replication [204]. Patino and co-workers conjugated a PNA targeting the TAR region of HIV RNA to TPP cation through a combination of carbamate and disulfide linkages (Figure 16B) [205]. The linker was stable in media containing 10% fetal calf serum for 48 h but was easily cleaved by glutathione treatment. FACS analysis showed 43% uptake of fluorescently labeled TPP–PNA conjugates in CEM cells in 6 h. The TPP–PNA conjugate inhibited replication of pseudotyped HIV-1 virions in CEM cells with IC_50_ 1.0 μM, while unmodified PNA was inactive. The TPP–PNA conjugate was not toxic at 2 μM [205].

Taylor and co-workers evaluated the splice correcting activity of PNA–R_9_ with additional conjugation of lipids and phospholipids at the N-terminus, such as, L–PNA–R_9_, P–PNA–R_9_, LP–PNA–R_9_, and LSS–PNA–R_9_ (Figure 16C) [206]. In HeLa pLuc705 cells, L–PNA–R_9_, LP–PNA–R_9_, and LSS–PNA–R_9_ showed similar bioactivity in the 1–3 μM range while PNA–R_9_ and P–PNA–R_9_ showed very little activity. The activity increased in the presence of 100 μM chloroquine suggesting that endosomal entrapment was limiting the efficiency [206]. A disadvantage of these lipid constructs was significantly higher toxicity compared to PNA and PNA–R_9_. The LC_50_ values for LSS–PNA–R_9_, L–PNA–R_9_, and LP–PNA–R_9_ were 3 (most toxic), 6, and 11 μM [206].

**Figure 17:**
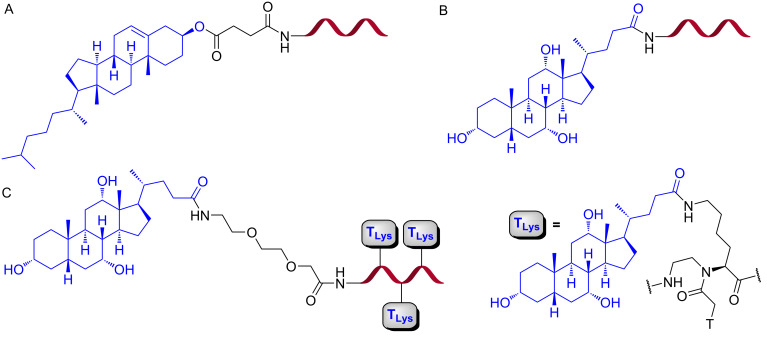
Structures of (A) chloesteryl–PNA, (B) cholate–PNA and (C) cholate–PNA(cholate)_3_.

Nielsen and co-workers conjugated cholesterol or cholic acid at the N-terminus of PNA (Figure 17) targeting a cryptic splice site in pre-mRNA in HeLa pLuc 705 cell line [207]. The conjugates were inactive in the splice correction assay when administered alone in up to 1 μM concentration. In contrast, both cholesterol and cholic acid PNA conjugates exhibited nanomolar antisense activity (EC_50_ = 25 nM, as measured by qRT-PCR) when delivered in the presence of lipofectamine2000, which was several-fold higher than the activity of PNA delivered by the DNA/lipid co-transfectant strategy [207].

**PNA delivery using receptor-specific ligands:** Corey and co-workers conjugated eight lactose moieties at the N-terminus of PNA targeting human telomerase and demonstrated cell-specific uptake of the Lac_8_–PNA conjugate in HepG2 cells that expresses surface bound asialoglycoprotein receptor (ASGPR). The addition of eight lactose moieties did not interfere with PNA’s binding to the target. In HepG2 cells, the Lac_8_–PNA conjugate linked through a cleavable disulfide bond was more active in inhibiting cellular telomerase (IC_50_ = 6 μM) than the conjugate linked by a stable amide bond (IC_50_ = 20 μM) [208]. However, the activity was still 50-fold lower compared to PNA delivered by the DNA/lipid co-transfectant strategy [209]. The Lac_8_–PNA conjugate having mismatched PNA or PNA conjugated to eight maltose moieties showed no activity at 20 μM [208].

**Figure 18:**
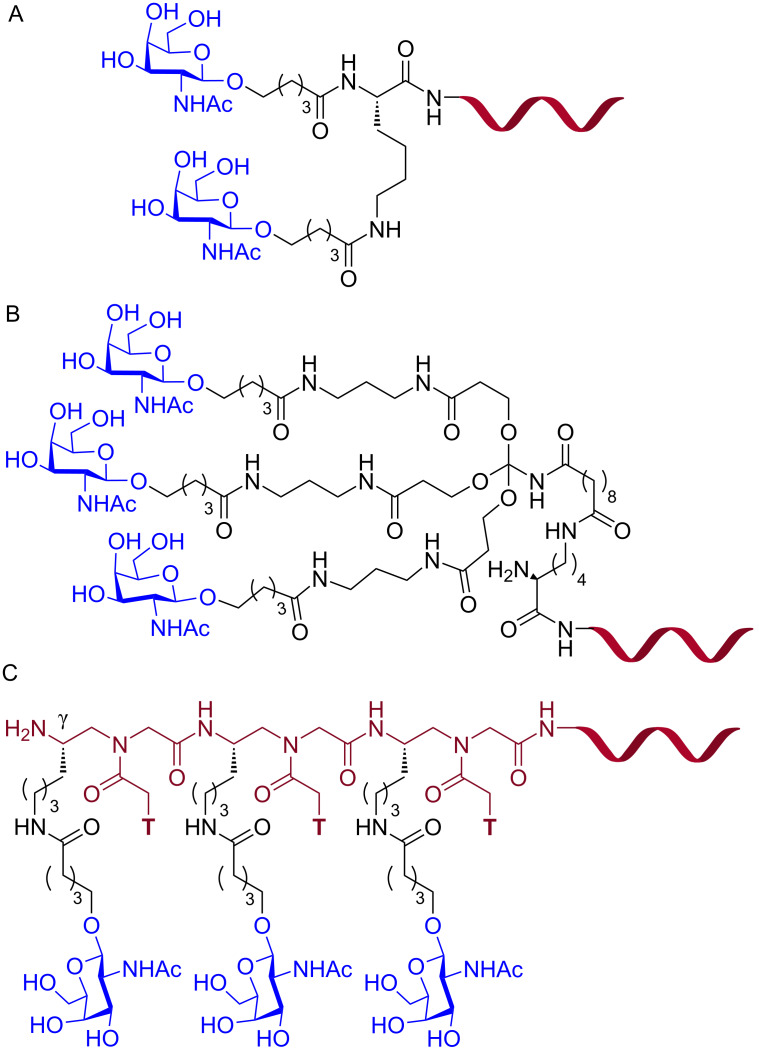
Structures of PNA–GalNAc conjugates (A) (GalNAc)_2_K, (B) triantennary (GalNAc)_3_, and (C) trivalent (T-γ-GalNAc)_3_.

Biessen and co-workers conjugated an antisense PNA targeting the human microsomal triglyceride transfer protein (MTP) to a bivalent (GalNAc)_2_K ligand (Figure 18), which has nanomolar affinity for the ASGPR [210], for receptor-mediated delivery of PNAs in hepatic cells [211]. In HepG2 cells, the antisense (GalNAc)_2_K–PNA at 100 nM concentration reduced the target huMTP mRNA levels by 35–40%, whereas no reduction was observed for scrambled PNA glycoconjugate and unmodified PNA [211]. A radiolabeled [^125^I]-(GalNAc)_2_K–PNA accumulated in parenchymal liver cells after intravenous injection in larger amounts than unmodified PNA (46% vs 3%). However, [^125^I]-(GalNAc)_2_K–PNA was rapidly cleared from the bloodstream with a plasma half-life of 0.38 ± 0.04 min [211]. In another study, (GalNAc)_2_K–PNA reduced MTP expression in mouse parenchymal liver cells by 70% [212].

Ganesh and co-workers, inspired by the recent success of siRNA-GalNAc platform [213–215], conjugated PNA to a trimeric GalNAc ligands, (GalNAc)_3_ and (T-γ-GalNAc)_3_ for receptor-mediated delivery to hepatocytes [216]. The triantennary (GalNAc)_3_–PNA conjugate (Figure 18B) at 4.0 μM specifically internalized in HepG2 cells that express ASGPR on their cell membrane, but not in Hek293 cells which lack ASGPR. Interestingly, the architecture of GalNAc conjugation to the PNA influenced the delivery. The trivalent (T-γ-GalNAc)_3_ having sequentially appended GalNAc units connected through the γ-carbons of the three T monomers (Figure 18C) showed 13-fold better uptake compared to a branched triantennary (GalNAc)_3_ unit (Figure 18B) (39% vs 3%) [216]. The GalNAc–PNA conjugates showed no cytotoxicity at 4.0 μM over 12 h; however, no in vitro antisense activity was studied [216].

**Figure 19:**
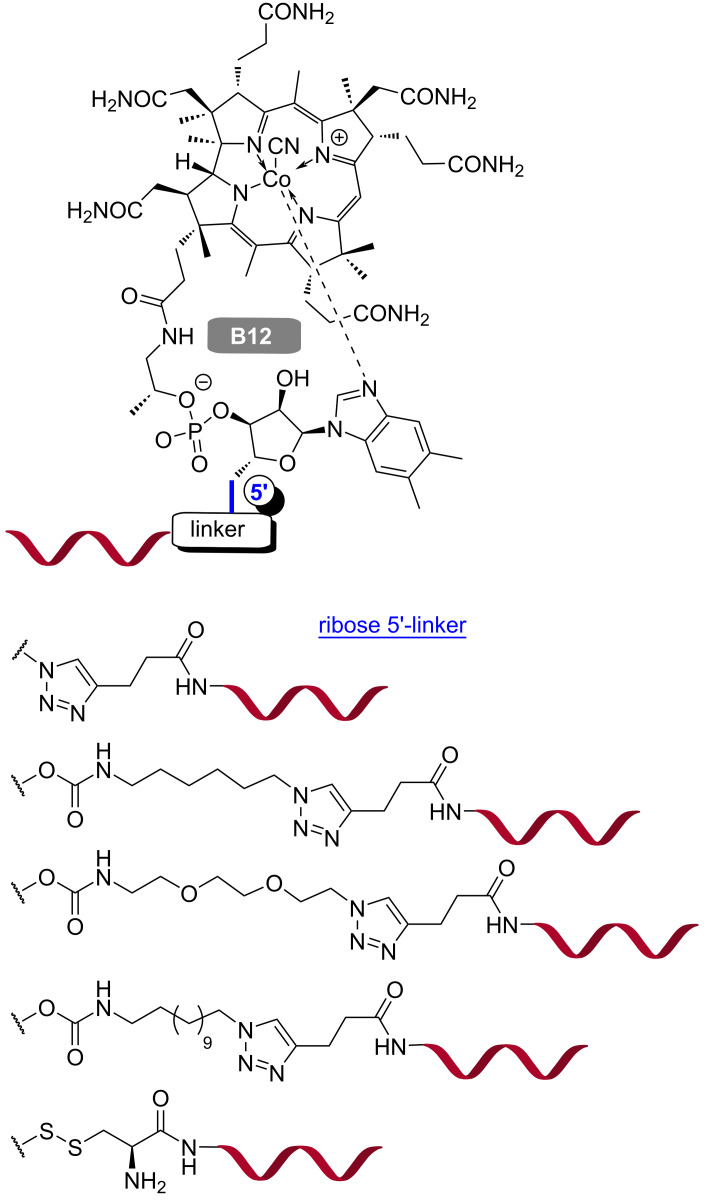
Vitamin B_12_–PNA conjugates with different linkages.

As mammalian cells are incapable of synthesizing vitamin B_12_, they have developed a well-established dietary uptake mechanism. Recently, the unique pathway of vitamin B_12_ absorption was used to deliver potential drug candidates, such as peptides and proteins, into the cells [217–218]. Gryko, Trylska and co-workers developed a synthetic strategy to covalently conjugate vitamin B_12_ (functionalized at the 5′-position of the ribose sugar) and PNA through a cleavable disulfide linkage (Figure 19) [219]. The same group synthesized a series of vitamin PNA–B_12_ conjugates with cleavable and non-cleavable linkers as well as various spacer length between PNA and B_12_. All conjugates were stable in bacterial Davis minimal broth and fetal bovine serum [220].

In *E. coli*, the PNA–B_12_ conjugates showed concentration dependent inhibition of *mrfp1* gene expressing a reporter red fluorescent protein, which was in contrast to the PNA–(KFF)_3_K conjugate that had constant activity of 70% over the 1–16 μM concentration range [220]. In *E. coli* the conjugates having the longest linker, PNA–(CH_2_)_12_–B_12_ and the shortest PNA showed slightly better uptake than PNA–(KFF)_3_K conjugate, while the opposite was observed in *S. typhimurium*. The PNA–B_12_ conjugate with a cleavable linker was the least effective in *E. coli*, whereas in *S. typhimurium* all PNA–B_12_ conjugates were equally effective. The activity differences in two bacterial cell lines highlighted the interplay between different bacterial cell walls and B_12_ in the membrane transport system [220]. Although the antisense effect of PNA–B_12_ and PNA–(KFF)_3_K conjugates was clearly demonstrated in the bacterial cells, it should be noted that both carriers reduced the binding affinity of PNA for the complementary RNA in cell-free systems [220].

In follow up studies, Gryko, Trylska and co-workers observed a similar antibacterial activity of PNA–B_12_ and PNA–(KFF)_3_K conjugates targeting the essential *acpP* gene in *E. coli* at 5 μM [221]. However, the bacterial growth inhibition activity of the PNA–B_12_ conjugates was media dependent in contrary to the PNA–(KFF)_3_K conjugates. Mueller Hinton broth (MHB) is a nutritionally rich medium where the receptors of vitamin B_12_ uptake on *E. coli* cell wall might have saturated, resulting in no antibacterial activity of the PNA–B_12_ conjugates compared to complete bacterial growth inhibition by the PNA–(KFF)_3_K conjugates. Changing the medium from MHB to Scarlet and Turner medium restored the bacterial growth inhibition activity of the PNA–B_12_ conjugates [221]. Most recently, Pienko, Trylska and co-workers reported that both B_12_ and B_12_-conjugates enter *E. coli* via the same route, a TonB-dependent unidirectional delivery through a recognition by the outer-membrane bound BtuB (vitamin B_12_-specific) receptor [222].

**Figure 20:**
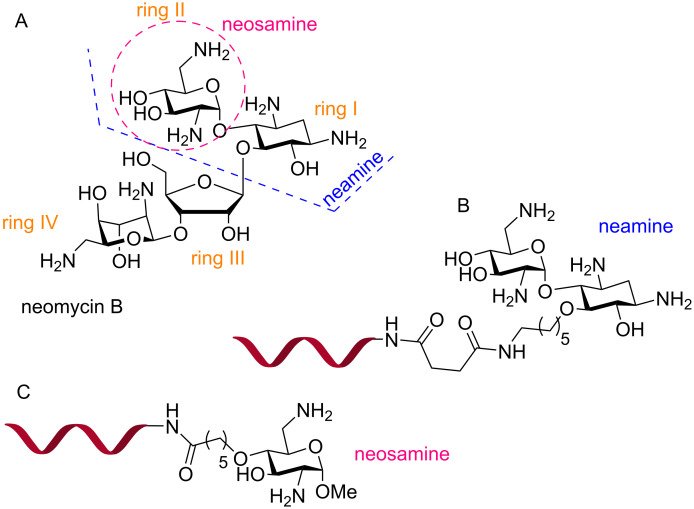
Structures of (A) neomycin B, (B) PNA–neamine conjugate, and (C) PNA–neosamine conjugate.

**PNA conjugates with cationic carbohydrate ligands:** Decout, Pandey and co-workers conjugated PNA with neamine (rings I and II of neomycin B, Figure 20A) [223–224]. The PNA–neamine conjugate showed improved water solubility and antiviral activity in CEM cells infected with HIV-1 carrying a reporter gene (IC_50_ = 1.0 μM). Interestingly, the PNA–neamine (Figure 20B) conjugate cleaved the target RNA sequence specifically [224]. In a later study, a PNA–neosamine (ring II of neomycin B, Figure 20C) conjugated through an amide linkage at the N-terminus of a PNA targeting HIV-1 TAR RNA performed even better than the PNA–neamine conjugate [225]. In CEM (T-lymphocytes) cells, 100% cellular uptake in the cytosol and nucleus of the PNA–neosamine conjugate at 0.3 μM was observed compared to 30% uptake of the PNA–neamine conjugate at 2 μM concentration [224–225].

The mechanism of uptake was studied in the Huh7.5 cells which have larger cytoplasmic space than the CEM cells. Unlike the delivery of PNAs using Tat and poly-arginine conjugates where a majority of the PNA conjugates were sequestered in endosome–lysosome compartments, the cellular distribution of PNA–neosamine conjugates was not affected by chloroquine co-treatment suggesting the absence of endosomal entrapment. No cytotoxicity was observed for the PNA–neosamine conjugates in the 0.2 to 1.0 μM range [225]. In CEM cells transfected with a reporter plasmid construct (pHIV-1 LTR-Luc), the PNA–neosamine conjugate at 0.5 μM and 1 µM inhibited Tat-mediated transactivation of HIV-1-LTR by 64 and 75%, respectively. The PNA–neosamine conjugates inhibited HIV-1 transcription in CEM cells infected with pseudo typed HIV-1 particles carrying a luciferase reporter with IC_50_ = 0.8 μM, without inducing cellular toxicity. Even at the concentrations as high as 100 and 500 μM, the PNA–neosamine conjugates had no negative effect on the cellular proliferation [225].

Kierzek, Chen, Prabhakaran and co-workers conjugated a triplex-forming PNA targeting the dsRNA panhandle structure of influenza virus with neamine [226]. As observed previously [224], the cellular distribution of the PNA–neamine conjugate in MDCK cells (Madin–Darby canine kidney) was homogeneous, including nuclei and mitochondria [226]. The PNA–neamine conjugate showed significant inhibition of viral RNA replication (IC_50_ ≈ 3 μM as measured by qRT-PCR) compared to a lack of inhibition with unmodified PNA. In another study, Chen and co-workers demonstrated that delivery of an antisense PNA–neamine conjugate in HEK 293T cells enabled splicing modulation comparable to the activity of the same antisense PNA delivered using a commercial X-tremeGENE 9 Transfection Reagent (both at 20 μM) [227].

Despite extensive research reviewed above, delivery of PNA is still an unsolved problem. Most of the PNA delivery systems have average EC/IC_50_ values in the range of ≈1–5 μM, with only a few reports of nanomolar activity. Increased cytotoxicity has been a limiting factor for most cationic peptides. Tat and (KKF)_3_K peptides are among the most common PNA-delivery reagents, most likely due to the balance between their ability to penetrate the membranes of various cell lines and synthetic accessibility. Common linkers to conjugate CPP and PNA are cleavable disulfides and stable amides, thioethers, or carbamates; the selection of linker becomes important based on the application, tissue/cell line, and mechanism of uptake of the CPP involved. Endosomal or vesicular entrapment and poor release remain as major reasons for the frequently observed micromolar activity of PNA conjugates. Therefore, the development and optimization of new non-endocytic delivery systems such as pHLIP, neosamine, vitamin B12, etc. or new peptides such as cyclic CPPs, etc. capable of efficient endosomal release might help realizing the full potential of PNAs for therapeutic and biotechnology applications. In conclusion, cellular uptake and in vivo delivery of PNA remains an area of active research where future developments hold promise for significant breakthroughs.

### PNA probes for research and diagnostic applications

PNA’s development has largely been guided by the biophysical limitations of PNA itself, specifically, its reduced solubility compared to native nucleic acids and poor cellular uptake. Early experiments were highly promising suggesting PNA binds nucleic acid targets with significantly higher affinity than analogous DNA/RNA probes. However, these experiments represented the potential of PNA under idealized conditions to bind to target compounds. Synthetic modifications, as previously discussed, have been extensively explored to translate these binding properties to applications in live cells, tissues, and living organisms where conditions are far from ideal. Many in vitro applications, where cellular uptake is not a concern and conditions are well controlled, use minimally modified PNA as the gold standard. Employing PNA in cells or tissues is more challenging, as the matrix becomes increasingly complex, more extensively modified PNAs are required to facilitate solubility and cellular uptake while maintaining selectivity. As a result, PNA has been found to have many applications as a research and diagnostic tool both in the lab and in the clinic [7–9], while advancement of PNA therapeutics, especially when compared to other nucleic acid derivatives [10–11], has notably lagged behind. To better understand the potential of PNA-based technologies, we will examine selected research and diagnostic applications highlighting the versatility of PNA as well as key limitations that hinder the extension of these technologies to therapeutic applications.

#### PNA-mediated PCR clamping

**Figure 21:**
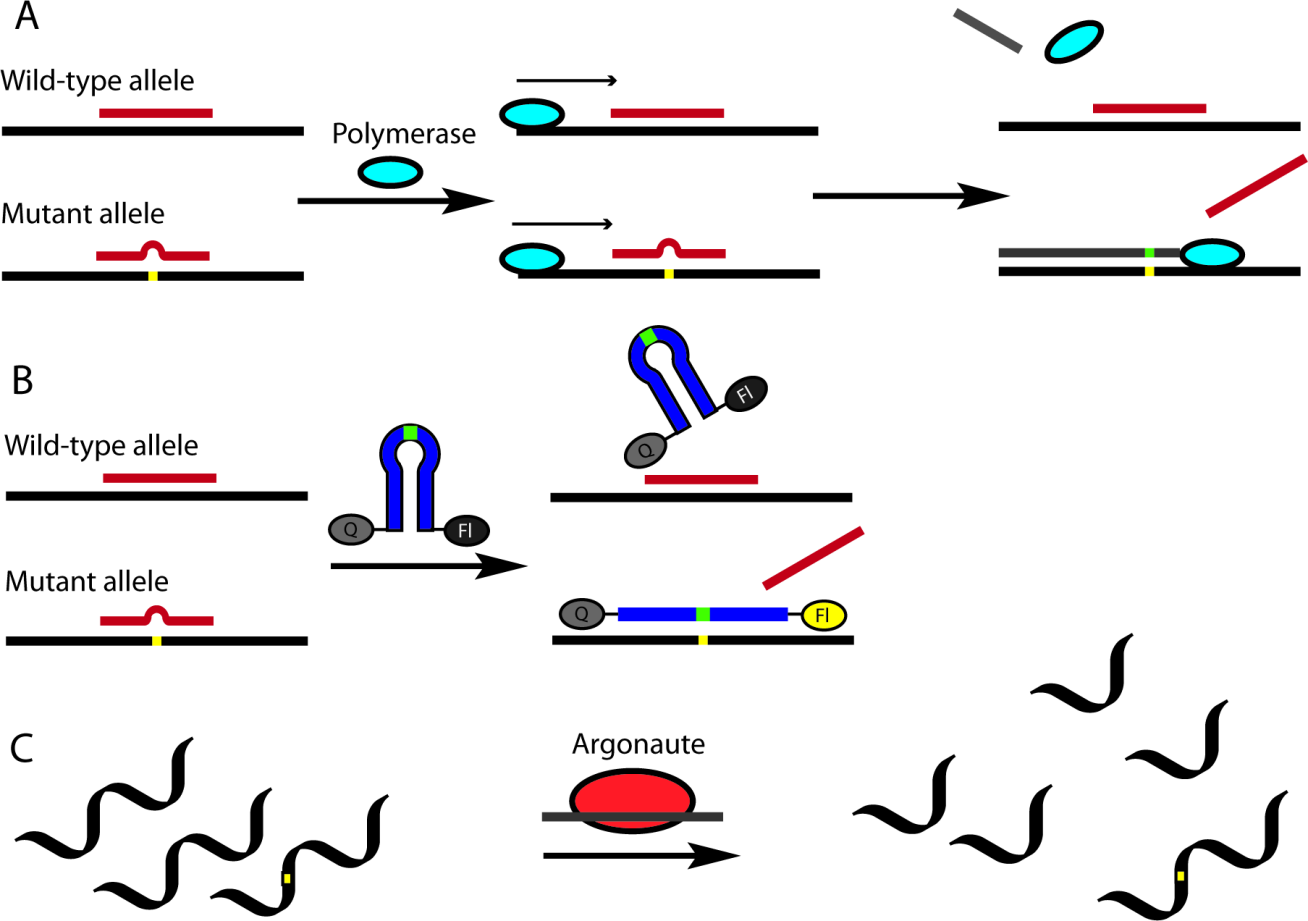
PNA clamp (red) binding to target DNA containing a mixture of sequences (A) PNA binds with higher affinity to the perfectly matched wild-type sequence while binding to the mutant containing as few as one mismatch is weaker. Once elongation begins, the perfectly matched complex stalls the polymerase inhibiting elongation while the mismatched complex dissociates allowing for elongation to continue; (B) LNA probes (blue) can also out compete PNA/DNA complexes mismatched allowing for sequence selective detection of mutant alleles; (C) NAVIGATER uses DNA-guided Argonaute to selective degrade wild-type oligos to enrich the mutant population increasing the sensitivity of PCR clamping.

The high specificity of PNA for target oligonucleotides in vitro was immediately identified as useful for PCR applications. One such approach, termed PNA-mediated PCR clamping, allows for selective PCR amplification of low population target sequences by suppressing the amplification of more abundant targets through PNA/DNA duplex formation. In the first report of PNA clamping (Figure 21A) the authors used 10- to 20-mer PNAs to suppress amplification of a plasmid DNA by clamping its primer [228]. A control plasmid without the target primer sequence showed no inhibition of PCR by the PNA clamps, demonstrating the sequence selectivity of amplification suppression. Clamping at or near the primer binding site was generally more effective while binding further from the primer gave effective clamping in two of the three PNAs tested. A single mismatch sufficiently destabilized the PNA–DNA duplex to allow for primer binding and selective amplification. This approach also worked using homopyrimidine PNAs that formed a PNA/DNA 2:1 triplex. This approach was extended to the detection of Ras proto-oncogene mutations [229]. A 15-mer PNA targeting codons 12 and 13 of wild type Ki-ras suppressed its PCR amplification. Mismatches between the PNA and mutant Ki-ras sequences resulted in lower stability allowing for 23-mer DNA primers to displace PNA turning on PCR amplification of the mutant sequences.

The ability to discriminate single-nucleotide polymorphism (SNP) in mixed populations makes PNA clamping especially useful in cancer detection [7]. Targeting epidermal growth-factor receptor (EGFR) mutations in non-small cell lung cancer revealed genetic heterogeneity in different lung cancer cell lines [230]. EGFR mutations can impact responsiveness to anticancer drugs, such as gefitinib. Clamping was done using 14- to 18-mer PNAs along with LNA molecular beacons (Figure 21B) to track the total amplification of different mutant subtypes. The mutated sequences were identified in the presence of 100 to 1,000-fold background of the wild-type EGFR. In total, 30 cell lines were screened by this method with 19 of those containing an EGFR mutation.

This approach was later integrated into a clinical application focusing on identifying mutations that make non-small cell lung cancer more susceptible to gefitinib [231]. A total of 132 patient biopsied tissue samples were analyzed at the Saitama Medical University Hospital with 34% being positive for mutations. A total of 29 exon 19 deletions and 16 exon 21 point mutations were detected by PNA clamping, all of which were confirmed by sequencing. The PNA probes displayed excellent sensitivity and selectivity, even for a mutant present at 1% with no false positives. Mutations in EGFR can also be detected in circulating free DNA from plasma [232]. Analyzing plasma samples is less invasive to patients making it an attractive alternative to biopsy sampling. Plasma samples from 60 patients were analyzed using PNA-mediated PCR clamping for mutations in exons 19 and 21 of EGFR. Of the 60 patients, 66.7% tested positive for EGFR mutations in the targeted exon. Of these, 70% were in-frame deletions in exon 19 and 30% were a specific arginine to leucine mutation in exon 21. Detection of mutants present in <1% in plasma samples, such as the T790M, remained a challenge. Sensitivity of PNA-mediated PCR clamping was recently improved by including DNA-guided Argonaute from *Thermus thermophilus* (*Tt*Ago) in an approach called NAVIGATER (Figure 21C) [233]. The DNA guide in *Tt*Ago corresponds to the wild-type allele for various genes (*KRAS, EGFR,* and *BRAF*). Prior to PNA-mediated PCR clamping, *Tt*Ago enriches either circulating free DNA or mRNA in mutant alleles by cleaving wild-type alleles complementary to the DNA guide. Sensitivity of PNA-mediated PCR clamping to mutations increased roughly 10-fold through this enrichment.

PNA-mediated PCR clamping directly applies PNAs high binding affinity and selectivity to silence an enzymatic process. Hybridization of PNA probes targets wild-type sequences to suppress their amplification with excellent selectivity and sensitivity blocking amplification based on a single nucleotide difference. While this is certainly impressive with clear implications in antisense and antigene applications PCR is an in vitro application that bypasses cellular uptake, which remains a significant roadblock to effective application of PNA in vivo. As the PCR application is in vitro, PNA already displays sufficiently high affinity and selectivity and therefore requires minimal improvements. Instead, most improvements in PCR technology have come from improved sampling methods either from a clinical standpoint (i.e., circulating free DNA detection) or from a biochemical standpoint (i.e., enrichment of low population species via NAVIGATER). Regardless, the application of PNA in PCR demonstrates both its selectivity and specificity as well as PNAs ability to impact enzymatic processes as a result of its strong binding.

#### Rolling-circle amplification

**Figure 22:**
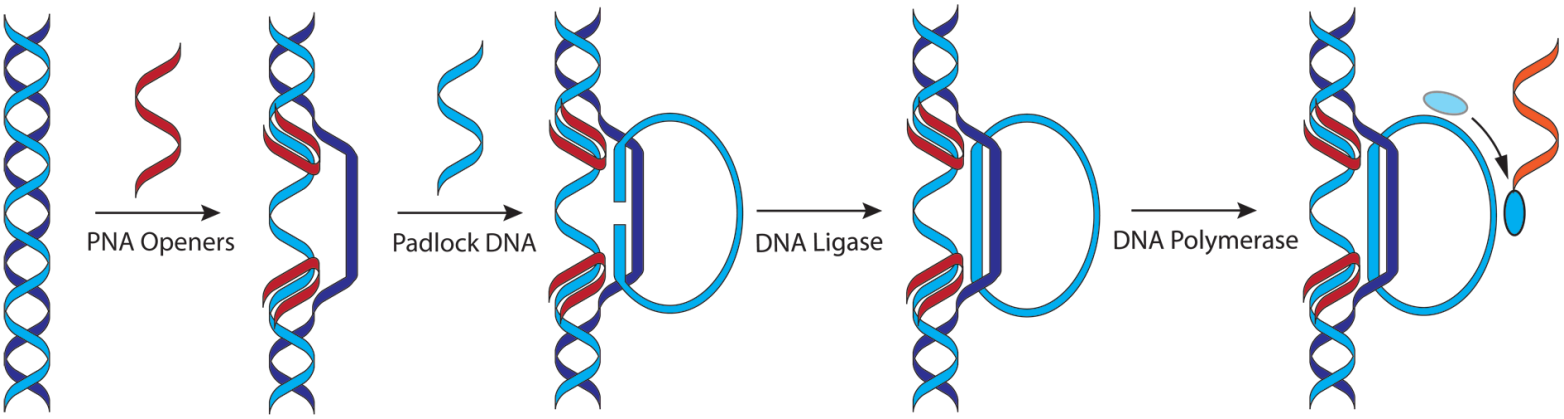
Rolling circle amplification using PNA openers (red) to invade a dsDNA target forming a P-loop. A padlock DNA probe (blue) can bind to the DNA liberated by the PNA openers. Ligase circularizes the padlock DNA resulting in an earring complex which acts as a primer for DNA polymerase. The resulting rolling circle amplification product (orange) can then be isolated or detected in solution.

PNA can also be used to liberate a target sequence from dsDNA using bis-PNAs openers to invade the double helix generating a P-loop ssDNA structure (Figure 2C). The liberated ssDNA can then serve as a hybridization platform allowing for oligonucleotide capture, topological labeling, or sequence-specific detection [234–235]. The ssDNA platform can also hybridize with so-called padlock probes to generate circularized oligonucleotides for rolling-circle amplification (RCA, Figure 22). After hybridization to the P-loop, the termini of padlock probes are fused by a DNA ligase generating an earring structure that acts as a primer for DNA polymerase resulting in the synthesis of long, repeating ssDNA which can then be detected. The PNA-mediated approach to RCA was first applied in topological labeling of dsDNA corresponding to the HIV-1 *nef* gene [236]. Two different DNA targets were used to determine the impact of topological constrain on RCA. One target was composed of a linear dsDNA fragment while the other was circularized forming a closed dumb bell structure. RCA proceeded smoothly despite of the geometric constraints of the dumb bell structure. While the kinetics of RCA were slower for the P-loops than for free ssDNA, signal generation still occurred quickly taking less than 90 minutes to reach its maximum.

PNA-mediated RCA has displayed a high level of sensitivity making it amenable to diagnostic applications. Detection of single-copy genomic DNA in *E. coli*, *B. subtilis* and *S. mutans* was accomplished using 8-mer bis-PNA openers and fluorescent probes targeting the ssDNA of the resulting P-loop [237]. A total of eight target sites were tested, all of which resulted in species-specific detection based on their unique P-loop sequences. This approach can be extended to targeting human chromosomal and mitochondrial DNA [238]. Multiple labels could be introduced by simultaneously targeting common 7-mer polypurine sequences flanking unique ≈20 nucleotide sequences. Chromosome specific padlock probes bound to each site specifically. Padlock probes also contained a shared sequence which was targeted by a sequence-specific fluorescent label allowing for visualization of multiple chromosomes with a single fluorescent probe. Chromosome specific labeling occurred for all targets with the main limitation being imaging sequences on sister chromatids with signals being distinguishable in only ≈30% of cases.

PNA can also be used as a capture probe in the design of microarrays for detection of genetic mutations. Recently, detection of mutations in *EGFR* was accomplished using RCA of the ssDNA of *EGFR* [239]. PNA complementary to the conserved 3′-end of the *EGFR* gene was covalently linked to the microarray through the N-terminus. As the target was ssDNA, no openers were required for padlock hybridization. Detection employed fluorescently labeled probes with graphene oxide acting as a quencher to increase sensitivity, which will be discussed in more detail in the coming sections. Selective detection of the mutant *EGFR* over the wild type was achieved using a species-specific padlock probe. Clear bands were observed down to 1 pM of the target sequence and was specific for the targeted mutation with the wild type generating no signal.

In RCA, the strong binding of PNA enables localized disruption of nucleic acid structure through invasion. The formation of P-loops is potentially useful for antigene and gene-editing technology with one major limitation. For simple PNA, low salt concentrations are required for invasion to occur that differs significantly from physiological conditions. Under physiological salt concentration, little invasion occurs as the dsDNA is stabilized making P-loop formation difficult. This is in part intrinsic to double-stranded oligonucleotide systems but can be partially remedied using more advanced PNA modifications. For example, replacement of pseudoisocytosine (J) in the triplex-forming portion of the clamp by 2-aminopyridine (M), which displays a higher binding affinity, may improve overall clamping efficiency [31]. The use of M as a partially cationic nucleobase may help counteract the stabilizing effect of salts on dsDNA affording potentially easier invasions.

The in vitro applications discussed above illustrate the power of PNA technology. The strong binding of PNA allows for suppression of enzymatic processes, such as PCR, and enable localized disruption of nucleic acid structure as demonstrated in RCA. PNA-mediate PCR clamping has been particularly impactful in diagnostic applications because of its efficacy and ease of application. The principle of disrupting either enzymatic processes or nucleic acid structure also has significant implications for PNA as a therapeutic. However, the biophysical limitations of PNA in cellulo and in vivo (i.e., low solubility, poor cellular uptake, etc.) have made the transition to antisense and antigene applications challenging.

#### Detection of DNA and mRNA

Imaging oligonucleotides using PNA is also widespread for in situ, in vitro, and in cellulo applications. The high binding affinity, sequence selectivity, chemical and enzymatic stability, and convenient functionalization makes PNA attractive for oligonucleotide sensing applications, such as fluorescence in situ hybridization (FISH) [8,240]. Fluorescent labeling of PNA is often operationally simple involving conjugation of dyes to the amino terminus, terminally attached amino acid residues, or functional groups of backbone-modified PNAs [241]. PNA-FISH was used to detect immunoglobulin kappa light chain mRNA in paraffin sections of fixed cells from tonsils using antibody-based signal amplification [242]. The immunoglobulin kappa light chain is one of the more abundant mRNAs in tonsil cells, making it an attractive first target. FITC-labeled PNA or DNA probes complementary to the target mRNA were hybridized in the sections of fixed cells. An anti-FITC antibody containing an alkaline phosphatase was then conjugated to the PNA/RNA duplex. After washing, treatment with 5-bromo-4-chloro-3-indolyl phosphate and nitro blue tetrazolium generated the observed signal through phosphatase-mediated enzymatic redox reaction. A similar amplification-based approach was used to detect HIV-1 in the cells of two AIDS patients in 2001 [243]. An N-terminally labeled FITC-PNA probe was designed to hybridize to the HIV protease gene. A horseradish peroxidase labeled anti-FITC antibody was then used to label the PNA. Next biotinylated tyramine reacts with the peroxidase, which is, in turn, labeled with horseradish peroxidase conjugated streptavidin. The cycle is repeated with the last step utilizing an Alexa Fluor 488 labeled streptavidin resulting in multiple Alexa Fluor 488 labels per hybridized PNA complex. Labeling occurred predominately in the nucleus, but some cytosolic labeling was also observed, possibly due to the presence of either HIV-1 DNA or RNA in the cytoplasm. Signal amplification is critical in generating a sufficiently bright enough signal for detection. Enzymatic signal amplification can be effective, but has limited applicability, as it often involves cumbersome antibodies and multiple rounds of amplification to generate a detectable response.

**Figure 23:**
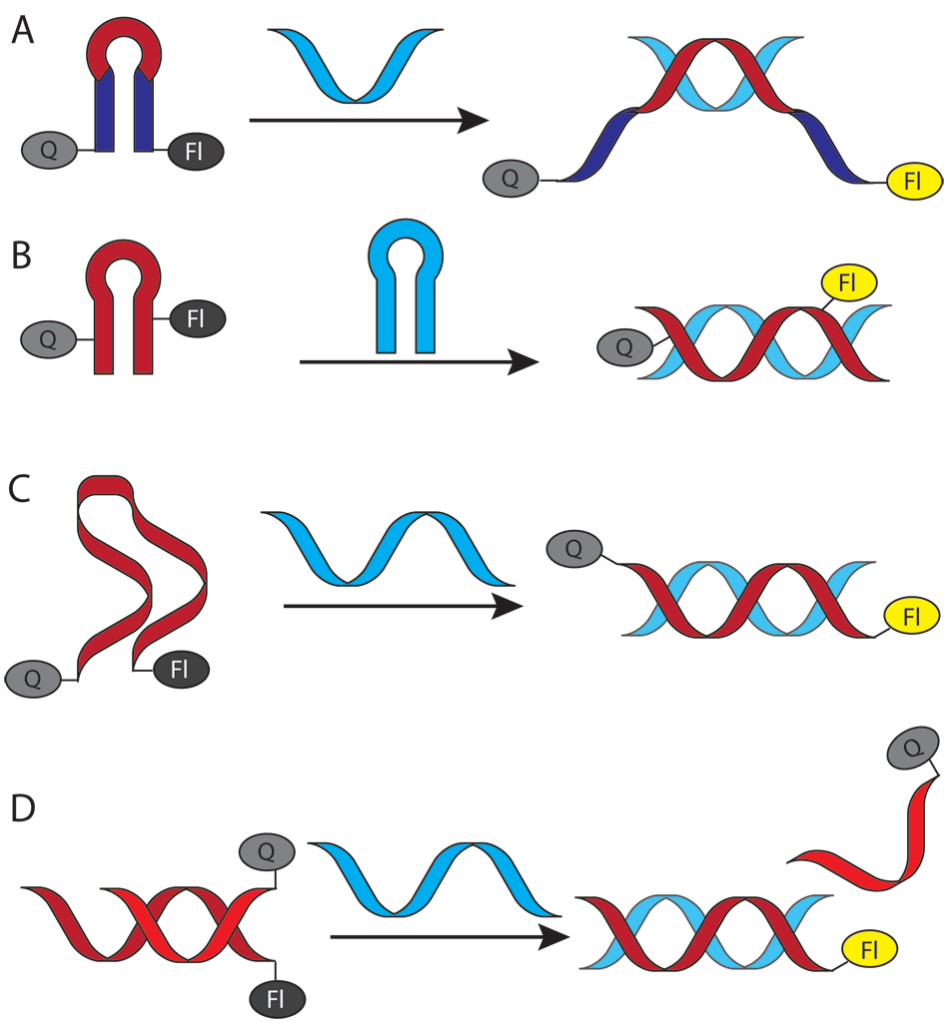
Molecular beacons containing generic fluorophores (Fl) and quenchers (Q) recognizing a complementary oligonucleotide. (A) PNA/DNA chimeras [244] (PNA in red, DNA in blue) and (B) PNA [245] with self-complementary stems were originally used to ensure close proximity of the fluorophore and quencher; (C) stemless beacons [246] lack partially self-complementary sequences instead relying on PNA aggregation to keep the fluorophore and quencher in proximity; (D) two complementary PNAs can also be used to ensure the proximity in dsPNA beacons.

Fluorogenic PNA helps address this limitation through the design of fluorescent systems which are somehow quenched in the absence of the complementary target sequence [241]. Several fluorogenic designs exist with molecular beacons being identified early as a means of increasing the sensitivity of PNA probes [244–245]. Due to sequence complementarity at the beacon termini, these probes form a hairpin structure in the absence of a complementary nucleic acid target referred to as a closed state. In the closed state, a fluorophore (Fl, Figure 23) and quencher (Q) are in proximity resulting in quenching of the fluorescence signal. Two different designs were reported in 1998. Lizardi and co-workers included 7-amino-4-methyl-3-coumarinylacetic acid (AMCA, FL) and 4-((4-(dimethylamino)phenyl)azo)benzoic acid (DABCYL, Q) modified T monomers in the last two AT/TA base pairs of their DNA/PNA chimera beacon (Figure 23A) [244]. Hybridization to the target sequence resulted in linearization of the PNA/DNA chimera probe enhancing fluorescence [244]. Schuster and co-workers replaced nucleobases with aminoacridine (Fl) and anthraquinone (Q) at proximal base pair positions in the middle of a PNA hairpin stem (Figure 23B) [245]. Titration experiments confirmed a 1:1 ratio between the probe and complementary dsDNA hairpins indicating PNA and DNA hairpins both open to form a PNA/DNA duplex.

Soon after, it was discovered that the stem portion of the design could be eliminated as PNA aggregation favored stacking interactions that quenched fluorescence in so-called stemless beacons (Figure 23C). Stemless PNA beacons binding either fully complementary or single-mismatched 16-mer ssDNA gave enhancement of the fluorescence signal [246]. An N-terminal cystine residue was modified with 5-((2-aminoethyl)amino)naphthalene-1-sulfonic acid (EDANS) which serves as the fluorophore while DABCYL-modified adenine acted as the quencher. The position of DABCYL impacted fluorescence enhancement with modification closer to the C-terminus giving ≈6-fold enhancement while modification closer to the middle of the sequence giving ≈4-fold enhancement. Stemless PNA molecular beacons were superior to stemmed PNA and stemless DNA molecular beacons when targeting ssDNA and dsDNA [247]. A fluorescein/DABCYL FRET pair attached to the termini of an 11-mer PNA displayed a rapid fluorescence response to ssDNA targets that was independent of salt concentration. The stemless DNA beacon also had a rapid response, but PNA had higher signal-to-noise ratio of ≈10. To target dsDNA, PNA openers were employed to generate a P-loop which acts as the hybridization platform for the PNA molecular beacon. Selectivity was modest with a matched-to-mismatched signal ratio of 1.5 at 25 °C, which increased to 20 at 46 °C.

**Figure 24:**
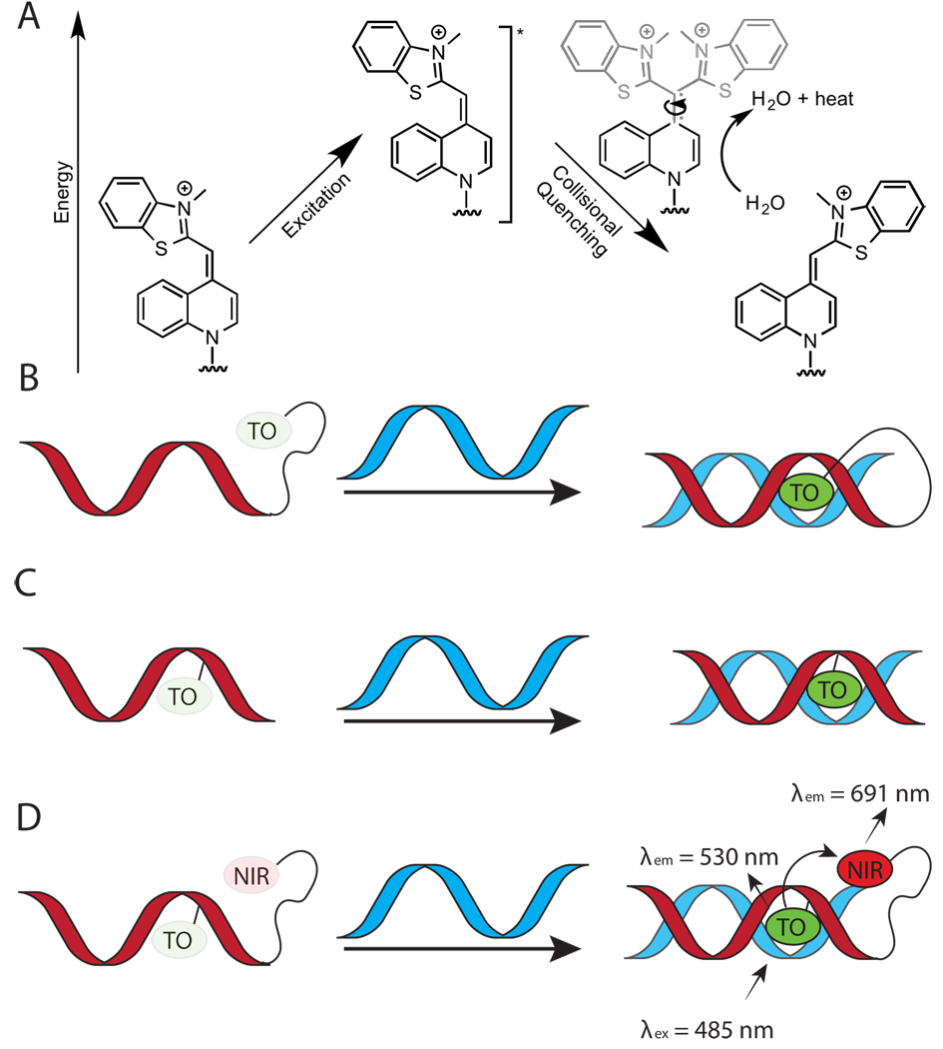
(A) Light-up fluorophores such as thiazole orange display fluorescence enhancement upon binding to a target oligo. In the free, single-stranded state, thiazole orange has a low fluorescence quantum yield as a result of collisional quenching with solvent upon excitation. (B) Thiazole orange can be tethered to PNA either at the terminus [151] or (C) through modified base pairs [150]. Modifying PNA at a nucleobase position with thiazole orange, typically referred to as forced intercalation (FIT) probes also results in sequence specific fluorescence enhancement. (D) FIT probes can be coupled in a FRET system with NIR-667 dye [249].

Another prominent approach in fluorogenic PNA probe design uses thiazole orange (TO) [151,248] or other cyanine dyes. These fluorophores display fluorescence enhancement on binding and intercalation in DNA, which eliminates non-radiative collisional quenching with solvent (Figure 24A). Early designs involved N-terminal labeling of PNA through a flexible linker allowing the dye to intercalate when the PNA probe was hybridized to a target oligonucleotide (Figure 24B) [151]. A 10-mer duplex forming PNA with a 10-atom linker displayed the greatest fluorescence enhancement of 45-fold (Φ_free_ = 0.0015, Φ_bound_ = 0.068). Homopyrimidine sequences had generally lower quantum yields (Φ_bound_ = 0.04–0.07) than mixed sequences (Φ_bound_ = 0.06–0.14). Ground state quantum yields varied significantly, likely because of different π-stacking interactions in the unhybridized probe. Kubista and co-workers applied a TO-PNA probe designed to detect a 1098 bp fragment of the *gusA* reporter gene [248]. A 10-mer polypyrimidine PNA using a 5-carbon linker to the quinoline ring of TO was designed to anneal at 67 °C, between the primer annealing temperature (54 °C) and the elongation temperature (74 °C), so the probe would not interfere with PCR amplification. This method displayed an excellent linear response over a large copy number range (R^2^ = 0.999, 300–10^9^ copies).

PNA probes having TO attached through a terminal linker showed promising light-up properties but exhibited significant signal variability depending on the sequence context. A more reliable fluorescence signal was achieved using a modified PNA monomer with TO serving as a nucleobase surrogate (Figure 24C), originally synthesized in 1999 by Seitz and co-workers [250]. While the TO nucleobase decreased PNA’s binding affinity compared to the fully complementary PNA/DNA duplex, the decrease was relatively minor (Δ*T*_m_ ≈1–3 °C) and showed little sequence dependency (± 1 °C when TO was paired opposite A, T, C, or G) [150]. Stacking interactions of TO helped stabilize PNA–DNA duplexes while simultaneously enhancing the fluorescence signal. The fluorescence response of TO was sensitive to the opposing nucleobase with fluorescence enhancement decreasing in the order of T > G > C > A.

Seitz and co-workers explored detection of single nucleotide polymorphisms using PNAs modified with the TO nucleobase [251]. To optimize these FIT-probes, attachment of TO through the quinoline or benzothiazole ring using linkers of various lengths (*n* = 1, 2, or 5) was tested in a 12- and 13-mer PNA against complementary 12- or 13-mer ssDNA. The FIT-PNA probe with the shortest linker attached to the quinoline ring had the highest sensitivity to mismatched base pairs adjacent to the TO nucleobase. Differences in melting temperatures ranged from 8 to 15 °C depending on the sequence as well as the position and identity of the mismatch. Fluorescence enhancement was 11–19-fold for fully-matched sequences while mismatched sequences only showed a 4–8-fold increase. Increasing the temperature increased mismatch discrimination.

FRET-based systems (Figure 24D) can use a single PNA containing intercalating nucleobases such as TO and a terminally tethered chromophore. Normally, this would be problematic as the background FRET signal may be high. When TO is used as a FRET donor, this is not the case, as fluorescence from TO is minimal in the unhybridized probe. Initial reports used NIR-667 conjugated to a lysine residue to serve as a FRET acceptor for TO [252]. Later studies significantly expanded the list of FRET pairs involving TO [253]. The presence of complementary DNA resulted in a 7- to 28-fold increase in TO fluorescence and a 15- to 89-fold increase in NIR-667 fluorescence. Several probes displayed significant enhancement in both TO fluorescence and FRET. One example using indotricarbocyanine (ITCC) separated by 10 nucleotides from TO gave a 452-fold enhancement in TO fluorescence on binding. Another example using NIR-664 separated from TO by 10 atoms displayed a 254-fold enhancement in FRET signal.

**Figure 25:**
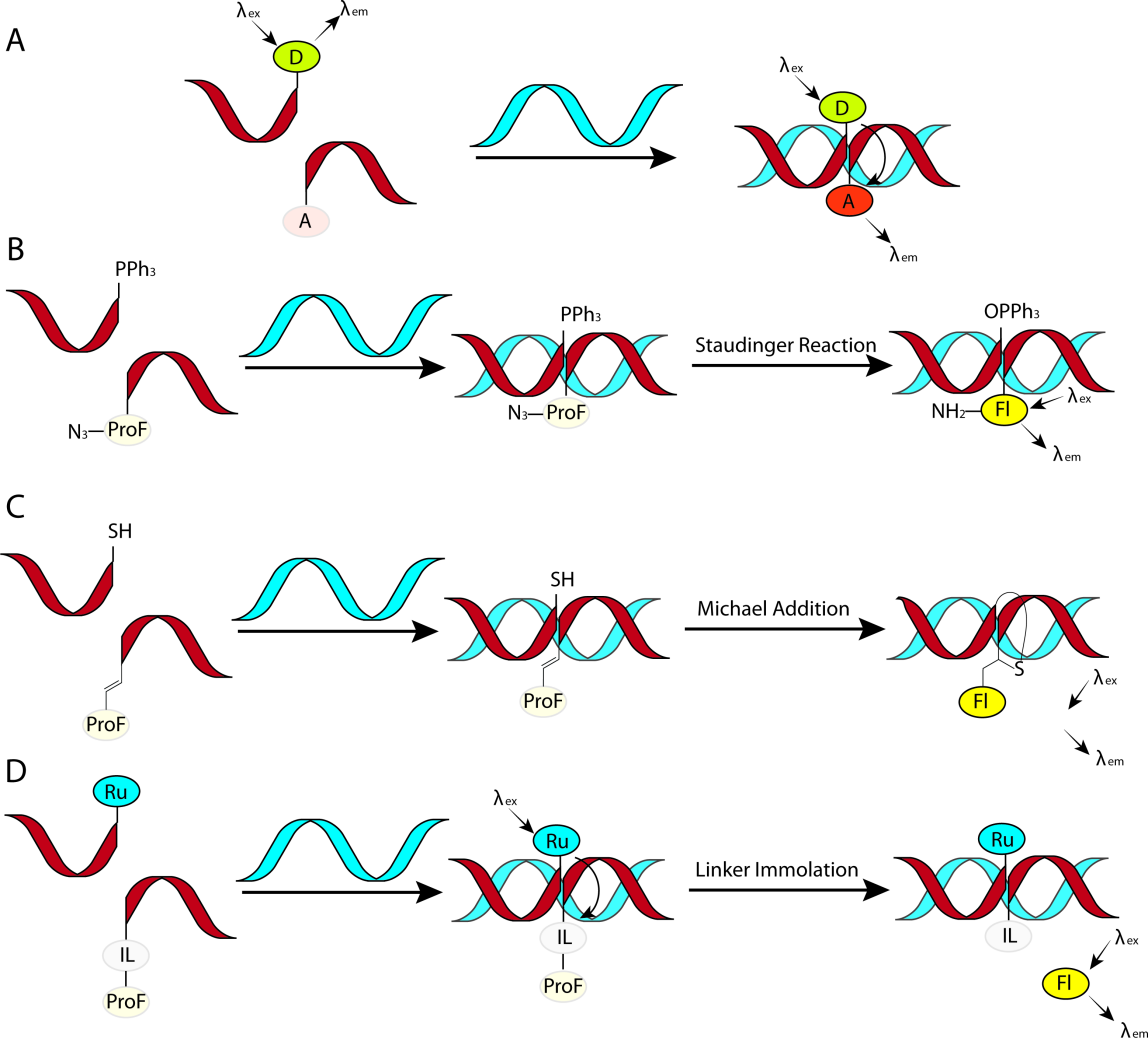
Templated fluorogenic detection of oligonucleotides using two PNAs. (A) Templated FRET depends on hybridization of PNAs to adjacent positions on the target sequence to bring the donor and acceptor in proximity. Templated reactions such as (B) Staudinger reaction or (C) conjugate addition of thiols can be used to turn on fluorescence of a caged pro-fluorophore. (D) Photochemical templated reactions target an immolative linker which both tethers and quenches a pro-fluorescent molecule.

Intron splicing of mRNA can be monitored using two labeled PNAs in a similar FRET-based detection method. This method employed two PNAs using TO and Alexa-594 to target the *RPS14A* gene mRNA [249]. In the unspliced pre-mRNA, these two PNAs are separated by >300 nucleotides. Upon splicing, this distance is shortened to 6 to 12 nucleotides, increasing FRET efficiency (Figure 25A). Using TO as a FRET donor can be exceptionally useful as the FRET signal is dependent not only on distance but also on intercalation of TO. Similarly, Artero and co-workers used Cy3/Cy5 labeled PNAs as FRET-based probes to visualize lymphocyte antigen 6 pre-mRNA which has two isoforms resulting from mRNA splicing in HeLa cells [254]. One N-terminal Cy5-labeled PNA was used as the acceptor with different Cy3-labeled PNAs acting as donors. The Cy3-labeled PNAs targeting either the spliced form or unspliced form of the mRNA displayed the expected FRET response on mRNA splicing.

Templated fluorogenic reactions use similar principles as FRET-based probes in that two probes with terminal labels are designed to hybridize in proximity on a target strand. Unlike simple FRET, a chemical reaction occurs due to the proximity of the labels, which produces a fluorescent signal [255]. Early efforts used Staudinger reaction to liberate amino groups from azido-modified fluorophores such as azidocoumarin or azidorhodamine (Figure 25B) [256–257]. The coumarin-based templated reaction used a C-terminal 7-azidocoumarin label and an N-terminal triphenylphosphine modification [256]. The reaction gave excellent fluorescence turn-on using two 8-mer PNA probes targeting an 18-mer ssDNA target. As little as 1% of the matched template generated a fluorescence response. In the presence of 20% template, single mismatches were easily discriminated with <5% conversion after ≈40 minutes compared to >30% conversion in the same time frame for the matched sequence. A 10-fold increase in fluorescence was observed using a catalytic amount of matched template DNA after only 15 minutes. The method was extended to visualize mRNA of O-6-methylguanine-DNA methyltransferase (MGMT) in HEK293 cells using cell-permeable GPNAs (Figure 5) having azidorhodamine and tris(2-carboxyethyl)phosphine (TCEP) modifications [257]. Incubation times were relatively short, less than 90 minutes, with the templated reaction showing similar fluorescence enhancement and mismatch discrimination as the azidocoumarin system.

Detection of mRNA in cellulo is more restrictive than detection in vitro requiring careful consideration of probe biophysics. FIT-PNA probes using TO, oxazole yellow (YO), and benzothiazole orange (BO) are ideal in this application as the fluorophore is relatively small and cationic limiting any negative impact on solubility. FIT-PNA probes enabled multichannel detection of influenza mRNA in MDCK cells [258]. In this study, 14-mer PNAs with TO outperformed PNAs with YO and BO in photophysical response at 25 °C displaying a 16-fold enhancement compared to 3.4-fold for YO and BO. At 60 °C all dyes performed admirably giving 34-, 15-, and 27-fold enhancements, respectively. Two probes, TO-FIT probe for neuraminidase (NA) and BO-FIT probe for matrix protein 1 (M1) were used for qRT-PCR as well as in-cell imaging. Streptolysin O facilitated the uptake of FIT PNAs into MDCK cells, which were then infected with influenza A. Fluorescence from the two probes developed at different time points with the TO-FIT probe for neuraminidase generating signal at two hours post infection. The signal was initially localized in compartments identified as nucleoli and spread into the cytosol over time. Control cells generated no signal suggesting the localization was a result of mRNA distribution and not PNA compartmentalization. The BO-FIT probe for M1 generated diffuse signal throughout the cell starting at five hours post infection.

Fluorescence reporters exhibiting red-shifted emissions are generally desirable because autofluorescence is reduced and the lower energy light required for excitation is less damaging to cells. Bisquinoline (BisQ) is a cyanine dye similar to TO with red-shifted emission (λ_em_ ≈ 610 nm BisQ, λ_em_ ≈ 500 nm TO) [155]. BisQ FIT-PNAs targeting the mutated *KRAS* oncogene DNA or mRNA had exceptional brightness (quantum yields Φ_bound_ = 0.22–0.26) and showed selective fluorescence from Panc-1 cells (*KRAS* mutant) but not HT-29 or Bxpc-3 cells (*KRAS* wild type) [155]. The sequence context for BisQ fluorescence response has been examined thoroughly to help in the design of BisQ FIT-PNAs [259].

BisQ-modified PNAs have been used to detect KRT20 mRNA, which is over expressed in colorectal cancer, in live cancer cells [260]. An 18-mer BisQ-modified PNA displayed 20-fold fluorescence enhancement in the presence of the target sequence while a scrambled PNA sequence containing BisQ gave no fluorescence response. The red emission from BisQ allows for superior detection of mRNA in tumors as red light scatters less and can penetrate deeper in tissue. Using two-photon microscopy allows for excitation of BisQ using longer wavelengths of light further improving tissue imaging. Spraying tumors with a solution of BisQ FIT-PNA targeting KRT20 visualized the mRNA to a depth of 240-micron in tumor tissue.

Detection of genes and mRNA has driven a large number of innovations in PNA technology, specifically in detection methods. Moving from parafilm sections to in-cell imaging showed that PNA can progress from in situ applications to more complex biological systems. The shift of detection to fluorogenic designs significantly improved the technology for diagnostic and research applications. Specifically, the development of FIT-PNAs has greatly improved the consistency of fluorescence responses using simple modifications without compromising sensitivity. FIT-PNAs have also been applied to in imaging in cells and tissues representing a significant step forward in this technology. Templated reactions have also shown promise as a sensitive method of detection with excellent selectivity which could be adapted for potential sequence selective payload delivery. The main limitation in extending these types of systems to therapeutics is the 1:1 ratio of PNA to target oligonucleotide. While this is acceptable for many modern sensing applications, the non-catalytic nature of silencing puts PNA/DNA or PNA/RNA duplex-based systems at a distinct disadvantage to enzymatic methods such as CRISPR/Cas9 or short interfering RNA (siRNA).

#### Detection of ribosomal RNA

The stronger affinity of PNA for RNA compared to DNA as well as the abundance of ribosomal RNA (rRNA) in cells makes rRNA targeted PNA a powerful diagnostic tool. Initial reports targeting rRNA focused on *Mycobacterium tuberculosis* complex (MTC) which is a genetically related group of bacteria responsible for tuberculosis. Both the 16S and 23S ribosomal subunits of several mycobacteria were screened to find partial sequence alignments specific to two members of MTC: *M. tuberculosis* and *M. bovis*. N-terminal FITC-conjugated 15-mer PNAs at 25–100 nM were shown to selectively target rRNA sequences specific to MTC complex or other mycobacteria [261]. After this initial report, both bacterial [262] and fungal infections [263] were identified from blood culture tubes using rRNA targeting PNA probes. Both publications used 15-mer FITC-conjugated PNAs targeting either the 16S rRNA of *Staphylococcus aureus* or the 26S rRNA of *Candida albicans* in clinically relevant samples*.* A total of 48 clinical isolates of *S. aureus* produced only one false-positive for *Stomatococcus*. Testing of 87 clinical blood culture specimens gave a 97% true positive rate and a 100% true negative rate [262]. For *C. albicans*, this technique had 100% sensitivity and specificity in samples of 148 clinical isolates and 33 real yeast-positive clinical blood cultures [263]. Both tests were fast and accessible, taking only 2.5 hours to obtain a potential diagnosis using techniques common in microbiology labs.

Raskin and co-workers imaged rRNA in fixed *E. coli* cells using a 16-mer stemless PNA molecular beacon with C-terminal DABCYL and N-terminal FITC labels [264]. This work compared the PNA beacon with an analogous 24-mer stem-containing DNA beacon. The DNA probe at 50 nM showed a linear response of fluorescence intensity depending on concentration of extracted target rRNA down to 12.5 nM of target rRNA, while the PNA probe’s linear response extended down to 0.39 nM. Both probes showed selective staining of rRNA in *E. coli* and *M. acetivorans*, but the PNA beacon was 3-fold brighter than the DNA-based probe. Signal intensity increased sharply during the first 15 minutes while reaching its peak at one hour while the DNA probe required several hours to generate a fluorescence response.

The exceptional sequence specificity of PNA along with high sensitivity and short time of analysis in imaging rRNA from blood cultures led to early development of commercial kits for PNA testing. Specifically, identifying *Candida* fungi has become increasingly important in determining course of treatment as different species of *Candida* respond differently to common antifungal drugs such as fluconazole. A multi-institute study comparing the *Candida* PNA FISH assay from AdvanDx with other routine tests showed that PNA FISH improved accuracy in microbe identification [265]. Similar results were obtained for PNA-FISH detection of different Gram-positive *Staphylococci* where accurate detection of *S. aureus* significantly improved the outcome for patients in intensive care [266]. In some instances, mutations in the rRNA sequence of bacteria or fungi may be associated with phenotypic changes such as antibiotic resistance. PNA-FISH is capable of identifying these mutant strains by directly targeting the rRNA mutation as demonstrated by the identification of clarithromycin-resistance in *Helicobacter pylori* [267]. Several different point mutations in the peptidyltransferase region in domain V of the 23S rRNA gene associated with the clarithromycin resistance were identified using 15-mer PNAs. These PNAs were labeled at the N-terminus with either Alexa Fluor 488 for the mutant rRNA or with Alexa Fluor 594 for the wild type rRNA. The PNAs were specific and sensitive to their target mutants and discriminated resistant and susceptible strains because of a single mismatch in the middle of the 15-mer PNA sequence.

Recent PNA probes targeting rRNA for clinical applications expand the scope of testing, improve the signal-to-noise, and reduce time of analysis. *Candida* QuickFISH BC from AdvanDx improves on their PNA-FISH kit for *C. albicans* [268]. Specific labeling for *C. albicans, C. galbrata, or C. parapsilosis* is done in multiplex using species-specific PNAs with different fluorescent labels*.* Quencher probes are then used to eliminate fluorescence from unhybridized PNA. Overall, the sensitivity was 99.7% and the specificity was 98.0% for the three strains of *Candida* targeted in this study. The time of analysis for this approach is only 30 minutes affording a fast and accurate diagnosis of multiple strains of *Candida* in one test.

Recently, a single-cell-based microfluidic detection of Gram-negative bacterial pathogens used molecular beacon PNAs targeting rRNA [269]. Two beacon designs, dsPNA beacons and stemless ssPNA beacons (Figure 23D), were compared. Cell lysates were incubated with PNA beacons at 25 nM to 200 nM followed by a quencher DNA sequence to eliminate fluorescence from any unhybridized probe. Probes were tested on four bacterial strains: *E. coli* (UPEC), *P. aeruginosa* (Pa127), *P. mirabilis* (Pm159), and *K. pneumoniae* (Kp128). The first two served as positive controls while the last two served as negative controls. Of these conditions, the dsPNA beacon at 25 nM had the highest signal-to-noise ratio and was species specific for *E. coli* and *P. aeruginosa*. Single cell experiments in 7 pL droplets using microfluidics confirmed results observed in bulk fluid analysis. The experiment aimed to seed 10% of droplets with bacterial cells. The dsPNA beacon resulted in 8% of droplets displaying fluorescence after 30 minutes compared to 1% of the droplets treated with the ssPNA probe suggesting faster hybridization of the dsPNA probe. Signal from bacteria-containing droplets compared to empty droplets was higher for dsPNA probes (≈3.4) to the ssPNA probe (≈2.2), suggesting that in no-wash applications, dsPNA beacons are superior to stemless molecular beacons in high-throughput diagnostics.

Diagnosis of bacterial and fungal infections is exceptionally accurate using PNA-based probes. The strong binding of PNA and the abundance of target rRNA has led to the development of commercial kits for disease identification. The simplicity and accuracy of these diagnostics has resulted in wide-spread adoption of this technique in clinical settings. While most PNA applications in rRNA sensing are limited to in vitro experiments, the strong binding of PNA to this critical component of cellular machinery make rRNA-targeting PNA therapeutics an attractive approach to treating microbial infections. The abundance of rRNA in cells, similarly to mRNA discussed previously, would likely be limiting to this technology as PNA binding and inactivating the rRNA would be non-catalytic and limited by the cellular uptake of PNA. However, development of therapeutic technology based on rRNA targeting with PNA may help supplement the physician’s toolkit as bacterial resistance to traditional antibiotics increases over time.

#### Detection of microRNAs

High binding affinity is critical for detection of microRNAs (miRNAs) because of their generally low copy number in cells and short sequence length (18–22 nucleotides). With miRNAs identified as increasingly prominent players in regulating gene expression, detection and quantification of these species is critical to deepening our understanding of miRNAs relation to disease. PNA-based fluorescence and electrochemical sensors of miRNAs have seen increasing use in a number of applications as highlighted in a recent review [9]. Early attempts at miRNA detection mirrored those of mRNA, using in situ enzymatic amplification to generate an optical signal [270]. Electrochemical detection using PNA for miRNA have also been explored with early reports using silicon nanowires [271]. As PNA lacks an intrinsic charge, this approach is well developed displaying excellent sensitivity.

Photochemically-induced templated reactions involving a [Ru(bpy)_2_phen]^2+^ catalyst have attracted significant attention due to ease of spatiotemporal control. In this two PNA templated system, one probe has an N-terminal rhodamine attached via an azide-caged immolative carbamate with the second probe containing a C-terminal [Ru(bpy)_2_phen]^2+^ group which can be excited with 455 nm light [272]. In the presence of a reducing agent, such as sodium ascorbate or NADPH, and the template sequence, excitation of [Ru(bpy)_2_phen]^2+^ results in azide reduction, which uncages rhodamine and generates a fluorescence signal. This process results in signal amplification as uncaged PNA dissociates and is replaced by another PNA still bearing the caged fluorophore. Backbone-modified (γ-CH_2_-OH, Figure 5) PNAs displayed the fastest reaction times and were sensitive to single mismatches when targeting ssDNA containing the sequences for either miRNA-21 or -31. Increasing the distance between the probes on the target sequence slightly decreased the efficiency of reaction, but ≈50% conversion was reported after 90 minutes, even when the PNAs were separated by 12 nucleotides. Templated reactions in BT474 cells and HeLa cells targeting miRNA-21 and -31 selectively showed fluorescence signal when using perfectly matched PNA, while a single mismatch in one of the two probes resulted in no observable fluorescence.

A FRET-based detection method using fluorescently labeled PNA along with nano graphene oxide referred to as PANGO have also been used to detect miRNAs [273]. Graphene oxide facilitates cellular uptake of PNA [273] while also quenching fluorescence via π-stacking [274]. This approach was used to target miRNA-21, -125b, and -96 with carboxy fluorescein (FAM), 6-carboxy-X-rhodamine (ROX), and Cy5 N-terminally-labeled PNAs. In all cases, a steady increase in fluorescence was observed up to 1,000 nM with a 1 pM detection limit. This approach could be multiplexed for miRNA detection in complex samples as no cross-reactivity was observed between the miRNAs and probes. The method did not show significant toxicity with a >90% viability in four cancer cell lines at ≤200 μg/mL of PANGO complex. While impressive, this approach lacks the signal amplification of templated reactions. RCA of miRNA synthesizing ssDNA with tandem repeats can be used along with PANGO complexes to increase detection sensitivity [275]. As discussed previously, RCA generates long, repeating ssDNA using a circularized padlock DNA probe complementary to the target oligonucleotide. In this case, the target miRNA-21, overexpressed in lung cancer patients, was normalized against miRNA-16. In the presence of graphene oxide, fluorescence of unbound PNA was completely quenched. The limit of detection was 0.4 pM for isolated miRNA and 0.7 pM when tested using total cellular RNA from A549 lung cancer cells. The method also worked in multiplex detection of miRNA-21, -31, and, -155 using three different FITC-, ATTO550-, and Cy5-labeled PNAs in a multi-well plate.

Fluorogenic coumarins can be uncaged using thiols through Michael additions that disrupt conjugation to a quencher. This was used to detect miRNA-132, -141, and -375 using PNA having C-terminal *n*-butyl thiol groups and PNAs having N-terminal styrene-quenched coumarin 334 [276]. The presence of DNA corresponding to the matched miRNA target gave a 15-fold increase in fluorescence intensity using two 7-mer PNA strands. Mismatches in the middle of the PNA probes significantly (>50%) decreased the fluorescence while mismatches close to the end of probes resulted in a modest fluorescence reduction.

**Figure 26:**
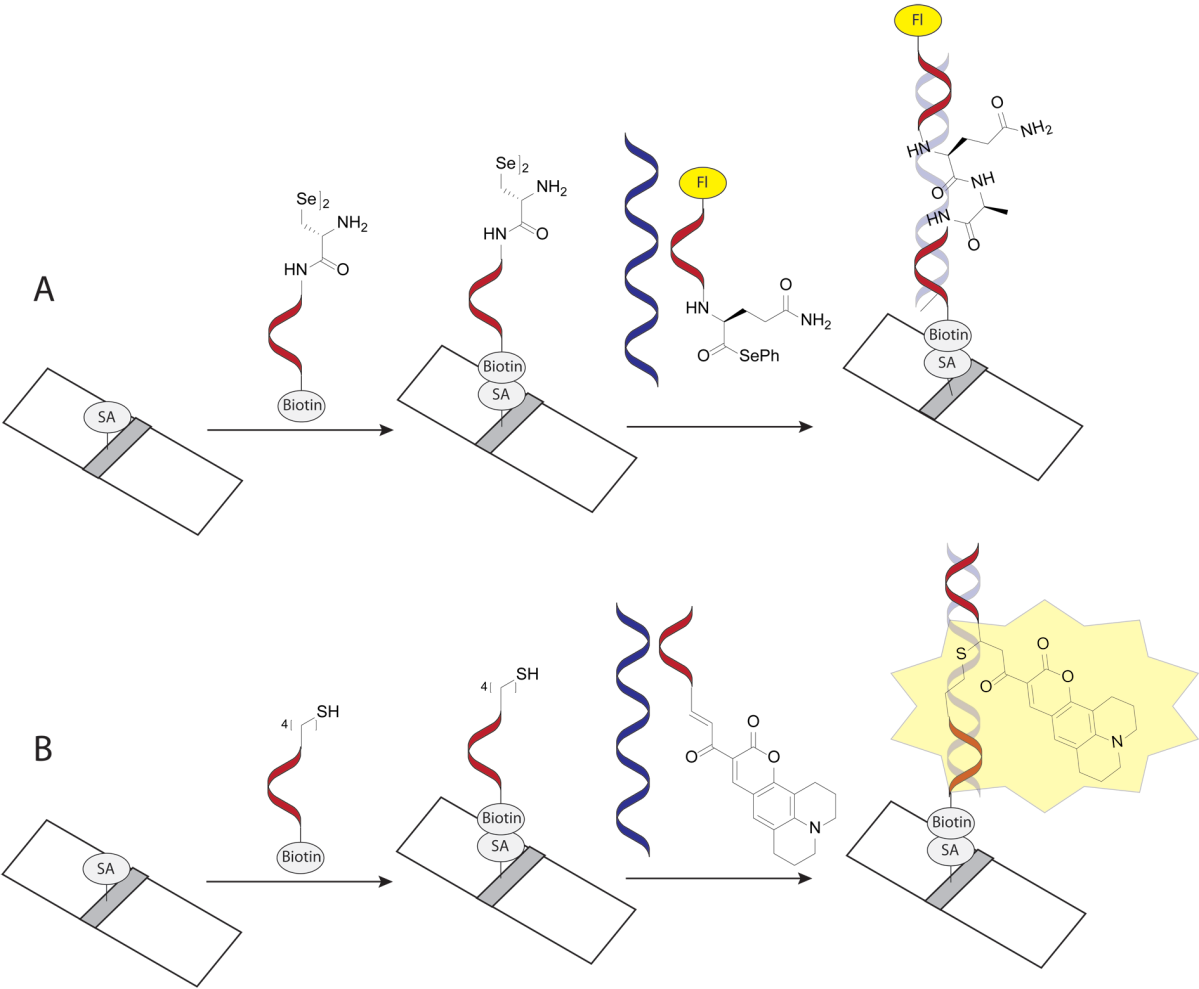
Lateral flow devices use a streptavidin labeled strip on nitrocellulose paper to anchor a capture PNA (red). The target oligonucleotide (blue) and the detection PNA probe (red) are then allowed to run the length of the strip. If the target is present, it will act as a hybridization scaffold bringing the two PNAs in proximity. This allows for either simple ligation (A) or fluorogenic ligation (B) which generates an optical signal allowing for detection of the target.

Lateral flow devices using PNAs have been developed for miRNA sensing. These devices often employ a similar design using a streptavidin-labeled lane in the middle of a strip of nitrocellulose paper which binds to a so-called PNA anchor through an N-terminal biotin label (Figure 26). Detection is then achieved through ligation to a separate fluorescently labeled PNA or through a templated fluorogenic reaction which forms a covalent linkage between the PNA probes [277–278]. Native chemical ligation is a well-established reaction involving a cysteine-mediated reaction of thioester to generate a peptide bond. A seleno-variant of this reaction was used in a lateral flow device and demonstrated a 10-fold faster reaction rate than the sulfur-based reaction (Figure 26A) [278]. This reaction was used to ligate two 9-mer PNAs with one containing a FITC-label. The limit of naked eye detection was <0.1 nM based on titration experiments of ligated PNA product. This method was then used to detect miRNA-31 and -21 in lysates from HeLa, MCF-7, and HEK293-T cells. HeLa cell lysates were positive for miRNA-31 and negative for miRNA-21, while MCF-7 cells gave the opposite result. HEK293-T cells were used as negative controls and, as expected, displayed no labeling. Another lateral flow device was developed by Ladame and co-workers using two 7-mer PNAs to detect miRNA-150-5p, which is a biomarker for preterm birth [277]. The two PNAs were connected through a templated Michael addition (Figure 26B). The detection limit was 9 nM with a linear correlation between signal intensity and target concentration between 10–200 nM. Plasma extracts from 18 patients tested using the lateral flow strip generated a statistically greater fluorescence signal (*p* value = 0.0006) from eight patients who delivered preterm than from the ten who delivered at term [277].

Targeting double-stranded pre-miRNA hairpins is also an effective sensing strategy as demonstrated by Winssinger and co-workers using two triplex-forming PNAs modified with [Ru(bpy)_2_phen]^2+^ and coumarin attached via an immolative pyridinium linker [117]. Sequence context proved to be important in maintaining selectivity for dsRNA pre-miRNA-31 hairpin, where longer PNAs (an 11-mer and 13-mer) showed some off-target fluorescence in the presence of ssRNA from the cleaved pre-miRNA, while shorter sequences (two 9-mer PNAs) were selective for dsRNA. A detectible fluorescence response was observed after 30 minutes in the presence of 12.5 nM pre-miRNA-31 [117]. Signal enhancement using this approach was as high as 20-fold.

Detection of miRNA is exceptionally important in the study of genetic diseases such as cancer. A number of miRNA biomarkers for disease and injury have been established and the ability to detect and quantify miRNAs with increasing sensitivity and precision will undoubtedly expand this list. Despite their relatively low abundance in cells, detection of biomarker miRNAs using PNA has developed rapidly as a viable diagnostic tool due to PNA’s strong affinity for RNA. PNA-based detection of miRNA has even been applied to potential consumer-friendly products, such as lateral flow devices. While many current applications focus on processed miRNA, targeting pre-miRNA is also a viable diagnostic approach. Developing methods for detection of both miRNA and pre-miRNA using PNA can help with understanding the role of miRNA in cells. Targeting these species has potential therapeutic implications as well, as the PNA–RNA complex may prevent processing of pre-miRNA or loading of miRNA into the RISC complex for mRNA silencing. The role of miRNA in coordinating cellular function through fine-tuning mRNA levels in cells makes it an attractive potential therapeutic target. Unlike mRNA or rRNA, the low copy number of miRNA and their broad effects means PNA-based silencing or attenuation of miRNA may have a strong impact provided the affinity of PNA is high enough. Hence, exploring anti-miRNA applications could be a fruitful area of research for PNA therapeutics.

#### Protein sensing

While PNA is typically designed to target nucleic acids, it can also be used to sense proteins. Hairpin peptide beacons function similarly to regular molecular beacons: they utilize a protein-targeting peptide sequence flanked on either end by short complementary strands of PNA to form a closed hairpin structure. Protein binding to the peptide sequence unfolds the structure giving fluorescence enhancement. Several proteins that bind short peptide substrates were targeted using this approach [279–281]. Src kinase is an important signaling protein that interacts with other proteins through its SH2 domain, which binds phosphorylated tyrosine residues on target proteins. A short peptide sequence from a known Src-SH2 binding protein containing phosphotyrosine served as the protein binding site for the probe. Two complementary 4-mer PNA sequences, terminated with pyrene-modified lysine residues that induced excimer/monomer fluorescence, closed the harpin. At 1 μM of peptide–PNA conjugate in the presence of 16 μM SH2-protein, a 10-fold increase in fluorescence was observed. Renin, an aspartic acid protease, was also targeted using a short renin peptide-inhibitor sequence and 4-mer PNA stems modified with NIR644 and DABCYL. At 100 nM of peptide–PNA conjugate in the presence of 120 nM renin, an 8-fold increase in NIR644 fluorescence was observed. Only a two-fold increase in the presence of 600 nM renin was observed from an analogous unstructured peptide probe. Another probe used a portion of HIV protein p17 and two PNA base pairs to form the closed structure quenching C-terminal BODIPY with N-terminal tryptophan [280]. This probe was then used to quantify anti-HIV antibodies that bind to the target peptide sequence in the probe. Unlike the previous report, PNA modification decreased the affinity of this peptide for its target from *K*_d_ ≈ 200 pM to 4 nM. However, a three-fold fluorescence enhancement and good emissivity allowed detection of anti-HIV antibodies down to 300 pM.

A similar approach was used recently to detect protein S100B, a known biomarker for brain trauma [281]. As protein expression is low in trauma victims and absent in healthy patients, a low nanomolar affinity is necessary for effective probe design. The peptide TRTK-12 served as the protein-sensitive portion of the probe as the peptide itself has a *K*_d_ ≈ 0.3 μM for the protein. Because S100B has two peptide binding sites, the best probe design contained two TRTK peptides connected through a peptide linker which also contained two G–C PNA base pairs. Using two TRTK peptides increased the probe affinity by two orders of magnitude (*K*_d_ ≈ 3 nM). Detection was achieved using lysine residues modified with either Alexa 488 or DABCYL in proximity with two G–C PNA base pairs to assure proximal fluorescence quenching. At 2 nM, the beacon generated 5-fold fluorescence enhancement in the presence of 80 nM S100B. Removing the PNA base pairs limited this enhancement to <1.5-fold [281].

While detection of proteins using PNA is relatively uncommon, the ease with which PNA monomers can be linked to peptides has been exploited in improving cellular penetration of PNAs for years. Using PNA base pairs to structure biologically relevant peptides therefore requires minimal adaptation of established procedures in peptide synthesis. The main strength of this approach is also its weakness, as PNA base pairs form strong interactions which help maintain the peptide in the closed state, but also hinder opening of the structure in the presence of the target protein. In spite of this, peptide beacons are useful in the detection of proteins with strong binding affinities for short target peptides. Similarly to PNA–peptide conjugates previously discussed, the combination of PNA with peptides and other biomolecules may lead to new or expanded applications of PNA both as research and diagnostic tools as well as potential therapeutics.

### Preclinical studies and attempts at therapeutic development

#### Demonstration of antisense and antigene potential

The potential of PNA for antisense and antigene applications was recognized almost immediately after its invention. Babiss and co-workers demonstrated that 10- to 20-mer PNAs could terminate both transcription and reverse transcription in vitro [38]. Nuclear microinjection of 15-mer or 20-mer PNA targeting SV40 T antigen mRNA reduced expression of the SV40 T antigen in 40% and 50% of injected cells, respectively. Similar results were obtained by Buchardt and co-workers two years later, showing that 10-mer PNAs arrested transcription of DNA under the control of T3 and T7 promoters [282]. Antisense properties of PNAs were explored more thoroughly in 1996 [283]. Both duplex and triplex formation with PNA could arrest translation of chloramphenicol acetyltransferase (CAT) mRNA both in vitro and in cell lysate. Inhibition using duplex forming PNA was limited to sequences immediately to the 5′ of the AUG start codon while targeting the coding region had little effect. Triplex formation using either two PNAs, bis-PNAs, or tail-clamp PNAs could arrest translation by binding either at the start codon or within the coding region of the mRNA. Corey and co-workers further explored PNA’s ability to inhibit translation by using 27 PNAs to target 18 different sites in a luciferase mRNA [284]. Duplex-forming PNAs targeting the terminus of the 5' UTR were found to be very effective (80%) in inhibiting translation of luciferase in COS-7 cells. Targeting other regions, including the start codon, was less effective. Sequence length also played an important role with 15- to 18-mer sequences giving <20% residual luciferase activity while a 10-mer sequence resulted in >85% residual activity.

#### PNA properties related to pharmacology

The high affinity and sequence specificity of PNA for natural nucleic acids [18–19] inspired multiple attempts to develop therapeutic approaches, such as antisense, antigene, and even more complex gene editing technologies [285]. PNAs form Watson–Crick duplexes with complementary DNA and RNA having significantly higher thermal stability and sequence selectivity (mismatch intolerance) than the natural DNA/DNA or DNA/RNA duplexes [3,286]. These favorable binding properties are critical for potency and selectivity of on-target pharmacological activity. Moreover, because of the entirely unnatural backbone, PNA is remarkably resistant to degradation by nucleases or proteases. In biological systems, PNA showed no significant degradation under conditions that completely cleaved various peptide substrates [287]. PNAs were stable in human and animal serums and eukaryotic cellular extracts under conditions where unmodified oligonucleotides had a half-life of only a few minutes [288]. Collectively, early studies clearly showed that PNA had impressive nucleic acid recognition potential and sufficient biostability for therapeutic applications. The most advanced examples of preclinical studies and cases were PNAs were tested in animal models in are summarized in Table 1 and discussed in more detailed below.

**Table 1:** Examples of advanced studies attempting therapeutic applications.

Disease or disorder	Mode of action	Target	Carrier, construct	Test model	Observed effect	Refs.

HIV	antisense	viral genome transactivation response element (TAR) in the 5′ long terminal repeat (LTR)	PNA– transportan (Figure 12)	HIV-1-infected H9 cells	inhibition of HIV-1 production	[289]
HIV	antisense	viral genome transactivation response element (TAR) in the 5′ long terminal repeat (LTR)	PNA–neamine (Figure 20B)	CEM cells infected with pseudo-typed HIV-1 S1 strain	inhibition of viral replication, cleavage of TAR RNA	[223]

hepatitis B virus (HBV)	antisense	terminal direct repeat (DR) sequence of pgRNA and mRNAs encoding HBV e antigen (HBeAg), core protein, x protein (HBx), and reverse transcriptase (RT)	PNA–Tat (Figure 12)	HepG2.2.15 cells and mouse model of acute hepatitis B	significant inhibitory effects against HBV replication in vitro and in vivo	[176]

malaria	antisense	PfSec13 mRNA essential for parasite proliferation in human erythrocytes	PNA–K_8_	parasites modified with luciferase reporter gene	dose dependent inhibition of parasite proliferation	[290]

duchenne muscular dystrophy (DMD)	antisense	spliceosomal skipping of exon 23 to restore correct dystrophin gene translation	PNA PNA–Tat PNA–MSP^a^ PNA–AAV6 PNA–AAV8	Mdx mouse model of muscular dystrophy	dystrophin expression in a dose-dependent manner in the injected muscle	[291–292]
DMD	antisense	spliceosomal skipping of exon 23 to restore correct dystrophin gene translation	unmodified PNA	Mdx mouse model of muscular dystrophy	dystrophin expression at high dose 50–100 mg/kg	[293]
DMD	antisense	spliceosomal skipping of exon 23 to restore correct dystrophin gene translation	PNA-BEPO^b^	Mdx mouse model of muscular dystrophy	low levels of exon skipping and dystrophin expression	[294]
DMD	antigene	exon 10 of the dystrophin gene	unmodified PNA	Mdx mouse model of muscular dystrophy	3% of gene repair was observed in the injected muscle	[295]
DMD	antigene	exon 10 of the dystrophin gene	unmodified PNA	muscle satellite stem cells form mdx mice, transplanted after PNA treatment into injured mdx mice	increase in the number of dystrophin-positive fibers detected after six months following transplantation in muscle	[296]

thalassemia	gene editing	β-thalassemia-associated splicing mutation at IVS2-654	γ-miniPEG tcPNA–K_3_, donor DNA, PLGA–NPs^c^	β-globin/GFP transgenic mice	editing of the defective gene with low levels of off-target modifications	[297]

lymphoma	antigene	enhancer Eμ DNA sequence that controls c-*myc* oncogene over-expression	PNA–NLS	Burkitt’s lymphoma (BL) cells and human BL lymphoma cells introduced in mice	decreased tumor size	[298–299]
multiple myeloma	antigene	transcription start site of RAD51 gene	PNA–NLS	SCID mice with implanted rabbit bone segments	sensitized multiple myeloma cells to melphalan treatment	[300]
tumor (lymphoma, leukemia)	antisense	miRNA-155 overexpressed in many cancers	PNA–penetratin, PLGA–NPs	nude mice with subcutaneously injected tumors	reduced tumor growth and miRNA-155 levels	[301]
tumor (lymphoma)	antisense	miRNA-155 overexpressed in many cancers	K_3_–PNA–K, R_3_–PNA–R, PLGA–NPs	NSG mice with injected subcutaneously tumors (U2932 lymphoma cells)	reduced tumor growth and miRNA-155 levels	[302]
tumor (HeLa cells)	antisense	miRNA-210 upregulated in response to hypoxia in various cancer cells and almost all solid tumors	γ-miniPEG PNA, PLGA–NPs	athymic nude mice with injected subcutaneously tumors (HeLa cells)	reduced tumor growth	[303]
tumor (lymphoma)	antisense	miR-155 overexpressed in many cancers	PNA–pHLIP	Tet-Off-based mouse model expressing miRNA-155 Nude mice subcutaneously implanted with neoplastic B cells	inhibition of miRNA-155 in vivo, delayed tumor growth, suppressed metastatic spread	[181]
prostate cancer	antisense	miRNA-21 frequently upregulated in solid tumors	PNA–Tat (49–57)	murine prostate cancer model with metastatic bone tumors	reduced tumor growth and metastasis	[304]

antibacterial	antisense	*acpP* gene encoding the ACP protein responsible for fatty acid biosynthesis	(KFF)_3_K–PNA	intraperitoneal mouse model of *E*. *coli* infection	reduced levels of bacteria	[305]
antibacterial	antisense	*rpo*A gene encoding RNA polymerase α subunit	PNA–KFF, PNA–ANT, PNA–Tat, PNA–PXR, PNA–RFR	*C. elegans* infected with *Listeria monocytogenes*	complete bacterial clearance with PNA–RXR at 32 μM.	[306]

^a^Sequences of cell penetrating peptides: MSP – ASSLNIASSL; AAV6 – TVAVNLQSSSTDPATGDVHVM; AAV8 – IVADNLQQQNTAPQIGTVNSQ; NLS – PKKKRKV (nuclear localization signal), pHLIP – AAEQNPIYWARYADWLFTTPLLLLDLALLVDADEGT(CNPys)G; TAT – YGRKKRRQRRRP, GRKKRRQRRRPGC, RRRQRRKKR or GRKKKRRQRRRYK; KFF – KFFKFFKFFK, ANT – RQIKIWFQNRRMKWKK; RXR – RXRRXRRXRRXRXB, RFR – RFRRFRRFRRFRXB. ^b^A long-acting, injectable in situ depot forming technology based on diblock and triblock poly(ethylene glycol)-polyesters solubilized in a biocompatible solvent; ^c^poly(lactic-*co*-glycolic acid) nanoparticle.

#### Inhibition of HIV and hepatitis B virus

Transactivation response (TAR) element from 5’ nontranslated region of HIV-1 viral genome together with transactivator protein are essential for the initiation of viral replication [307]. Pandey and co-workers reported that anti-TAR PNA conjugated with transportan CPP inhibited transactivation of the HIV-1 LTR, resulting in decreased production of HIV-1 virions by chronically infected H9 cells [289]. Latter studies found that the mechanism of cellular uptake of the PNA–transportan conjugate was neither receptor-dependent nor endocytosis [308]. The PNA conjugate permeated the virus envelope and inactivated HIV-1 virions in the plasma prior to their entry into cells; hence, these conjugates could be envisioned as potential prophylactic agents to block HIV-1 infection following accidental exposure to the virus. In another study, the same sequence of PNA was conjugated to neamine (Figure 20B) which improved PNA solubility and cellular uptake. The PNA–neamine conjugate also enabled cleavage of target RNA thus enhancing HIV inhibition [223].

The terminal direct repeat (DR) sequence of hepatitis B virus (HBV) pre-genomic RNA plays an important role in the synthesis of the HBV genome. As discussed above, the PNA–Tat conjugate antisense targeting DR effectively inhibited HBV replication in vitro and in vivo, with potency similar to clinically used antiretroviral drug lamivudine [176]. This study suggested that PNA–Tat has potential for treatment of HBV infections.

#### Malaria

The PNA–K_8_ conjugate was explored as an inhibitor of malaria-causing protozoan *Plasmodium falciparum* [290,309]. To reach the target RNA in parasites at their intracellular blood stage, PNA should cross four membranes: the erythrocyte membrane, the parasitophorous vacuole, the parasite’s plasma membrane, and the parasite’s nuclear envelope. The PNA–K_8_ antisense effect was more pronounced when the conjugate was added in the trophozoite stage and 4.8 μM of the anti-Sec13 PNA–K_8_ conjugate downregulated PfSec13 expression by approximately 75% [290].

#### Duchenne muscular dystrophy

Duchenne muscular dystrophy (DMD) is an X-linked genetic disorder and the most common form of muscular dystrophy caused by mutations in the dystrophin gene that lead to essential shortage of the functional protein. Respiratory or cardiac failure caused by DMD usually become fatal before the end of the third decade of life. Antisense oligonucleotides have been shown to induce specific exon skipping and restore the correct reading frame and expression of functional dystrophin [294].

Wood and co-workers found that unmodified PNA and various PNA–peptide conjugates, including Tat, muscle-specific peptide (MSP), and adenoassociated virus functional domains AAV6 and AAV8 induced exon skipping and dystrophin expression in a dose-dependent manner after intramuscular injection in mdx mice [291]. Interestingly, this study observed no significant difference in potency of unmodified PNA and its peptide conjugates, which was attributed to peptides selected in this study not being sufficiently efficient in transfecting specifically in muscle and escaping endosomal entrapment. Examination of morphology of muscle cells treated with unmodified PNA or PNA–peptide conjugates by hematoxylin and eosin staining did not show local muscle toxicity [291].

Yin and co-workers explored the potential of PNA (various lengths from 20- to 30-mer) to induce exon skipping and expression of dystrophin by systemically administering PNA to mdx mice through weekly intravenous injections of 50–100 mg/kg, over the course of three weeks [293]. Enlarged number of dystrophin-positive fibers was observed in several tissues: abdominal muscle, gastrocnemius, and quadriceps, but not in the heart. The longest PNA (30-mer) caused more significant increase of dystrophin expression in tibialis anterior muscles than other shorter versions. However, an acidity-related toxicity was observed for PNA 30-mer, which may be related to difficulties in purification and solubilization of longer PNAs [293].

Brolin and Nielsen investigated the effect of in situ forming depot technology (BEPO, PEG-PLA biodegradable polymer) and PNA-oligonucleotide formulation in systemic administration of a 20-mer splice switching antisense PNA through intravenous and subcutaneous routes in the mdx mice [294]. Intravenous administration resulted in fast renal/bladder excretion of the PNA (half-life ≈20 min) while subcutaneous administration led to a 2–3 times slower excretion. However, due to biphasic kinetics, release of 50% of the PNA dose from BEPO–PNA formulation takes approximately 10 days. Overall, the PNA–BEPO administration did not significantly improve antisense activity [294].

Brolin and Nielsen observed lower dystrophin expression than that reported by Yin and co-workers [293]. Interestingly, Brolin and Nielsen observed PNA precipitation when the pH of PNA administration solution was adjusted above 4 at 1 mM concentration (required for dosing at 50 mg/kg), suggesting that acidity required to solubilize longer PNAs may have caused toxicity observed by Yin and co-workers [293]. Collectively, the PNA antisense agents targeting muscles, even in the case of compromised muscle fibers in muscular dystrophy, still need major improvements to become therapeutically relevant, regardless of the administration route and long-acting depot or heteroduplex formulation [294].

#### Thalassemia

Thalassemia is an inherited blood illness characterized by decreased hemoglobin production. As a monogenic disorder, β-thalassemia has been a focus of gene therapy efforts. Most notably, significant progress in gene editing of hematopoietic stem cells has been reported by Glazer’s team in collaboration with other groups [297]. Glazer and co-workers have been studying triplex-forming bis-PNAs as gene mutagenesis and editing tools for more than two decades [285,310–311]. Recently, addition of γ-miniPEG modification (Figure 5) to tail-clamp PNAs (tcPNA, Figure 2) increased the gene editing frequency of up to 6.9% in a thalassemic mouse model [297]. The gene editing construct included γ-miniPEG modified-tcPNA, conjugated with three lysines at each termini, and donor DNA, formulated in poly(lactic-*co*-glycolic acid) nanoparticles (PLGA-NPs), and was used together with stimulation of the stem cell factor (SCF)/c-Kit pathway. The use of γ-miniPEG modified-tcPNA gave almost double gene editing than unmodified tcPNA, presumably due to enhanced strand invasion and DNA binding because of the helical pre-organization enforced by the γ-miniPEG modification [89].

PLGA-NPs were previously used for systemic delivery of FDA-approved drugs and effectively delivered PNA/donor DNA combinations into primary human and mouse hematopoietic cells with essentially no toxicity [301,312–313]. For in vivo studies, PNAs and donor DNAs, at a molar ratio of 2:1, were incorporated into PLGA-NPs and administrated by intravenous injection while SCF was administrated intraperitoneally 3 h before PLGA-NP injections. Importantly, the overall off-target modification frequency in γ-miniPEG modified-tcPNA treated thalassemic mice was 0.0032%, which was 1,218-fold lower than the frequency of β-globin gene editing [297]. In addition, minimal immune or inflammatory responses were observed in this study according to cytokine array analyses. The combination of nanoparticle delivery, γ-miniPEG modified-tcPNA, and SCF treatment can be basis for a minimally invasive cure for genetic disorders that can be achieved simlpy and safely by intravenous and intraperitoneal administration [297]. About 4% frequency of gene editing in total bone marrow cells achieved in the thalassemic mice was adequate to achieve a clear improvement in phenotype. Higher editing frequencies have been achieved in cell culture carrying the same thalassemia-associated β-globin mutation using TALENS (33%) and CRISPR/Cas9 (12–16%) [314–315]. However, direct comparison of PNA with TALENS or CRISPR/Cas9 is not possible because the studies used different cell lines and data analysis methodologies.

#### Anticancer PNAs

PNAs have been explored as antigene and antisense agents against various types of cancer (Table 1). Boffa and co-workers reported that a PNA–NLS conjugate (18-mer) complementary to intronic Eμ enhancer DNA sequences, inhibited the expression of the c-myc oncogene under the Eμ enhancer control in Burkitt’s lymphoma (BL) cells and human BL lymphoma cells introduced in mice [298–299]. After injection in mice, PNA reached the maximum concentration in the tumor in 90 minutes, with less accumulation in kidney, liver, spleen, heart, and brain. PNA was present in tumors for at least 600 minutes at a concentration that effectively inhibited BL cell growth in culture [299]. Short-term or long-term toxic effects were not observed. The tumor volume started to plateau after eight injections of PNA–NLS. Necrosis (about 8% of the neoplastic cell) was observed in histology of the tumor of PNA-treated mice [299]. Reis and co-workers reported that PNA–NLS targeting the transcription start site of RAD51, protein that mediated recombinational DNA repair and is overexpressed in multiple myeloma, sensitized multiple myeloma cells to melphalan treatment [300]. Melphalan is chemotherapy medication used to treat multiple myeloma, ovarian cancer, melanoma, and AL amyloidosis.

MiRNAs have been a well-established target for antisense anti-cancer approaches [316–317]. Fabani, Vigorito, and co-workers reported that antisense PNA conjugated with three lysines (K–PNA–K_3_) completely abolished the expression of miRNA-155 induced by intraperitoneal lipopolysaccharide (LPS) injection after dosed systemically at 50 mg/kg for two days [190]. Slack and co-workers reported that antisense PNA decreased miRNA-155 expression and tumor growth when injected as PLGA–NP formulations in nude mice carrying tumor cells from NesCre8 [301]. The PLGA–NPs were modified by penetratin that is attached to the NP surface via a PEG linker. A single local intratumor injection of PNA–PLGA-NP at 1.5 mg/kg reduced tumor increase from 10-fold to 2-fold, while two intravenous injections (1.5 mg/kg) reduced tumor increase by ≈50% relative to control tumors. These decreases in tumor growth correlated with a decreased number of miRNA-155 per tumor cell.

Bahal and co-workers studied short PNAs (8-mers) targeting the seed region of miRNA-155 in NSG mice carrying tumors induced by subcutaneous injection of U2932 lymphoma cells [302]. PNA conjugates with lysine and arginine, K_3_–PNA–K and R_3_–PNA–R, were formulated with PLGA–NPs and delivered by tail vein injection. The PNA 8-mer showed similar and even better efficacy in reducing the tumor growth compared to full length PNA 23-mer; PNAs without additional amino acids did not bind to miRNA-155 and arginine conjugates were slightly better than lysine conjugates. The authors did not observe any signs of immune response or toxicity in histology of liver, kidney and other organs [302].

Glazer and co-workers showed that γ-miniPEG modified-PNA antisense to miRNA-210, an oncogenic miRNA that helps tumor cells to survive and proliferate under hypoxic conditions, significantly delayed growth of a human tumor xenograft when administered by intratumoral injection in mice using PLGA–NPs [303]. The γ-miniPEG modified-PNA was significantly more active than unmodified PNAs. However, intravenous administration of the PLGA–NPs/PNA was not effective in preventing the tumor growth. In another study, Slack and co-workers showed that antisense PNA–pHLIP conjugate (Figure 13) showed significant survival advantages in nude mice subcutaneously implanted with neoplastic B cells compared with a commercially available locked nucleic acid antimiR optimized for in vivo miRNA-155 silencing [181]. The PNA–pHLIP conjugate delayed tumor growth and suppressed the metastatic spread of neoplastic lymphocytes to other organs, without causing toxicity in healthy mice.

Youn and co-workers compared antisense PNA and locked nucleic acids (LNA) conjugated with a shorter version (amino acids 49–57) of the Tat peptide and targeting miRNA-21 murine prostate cancer model [304]. This study found that PNA conjugates showed better stability and efficacy than LNA conjugates with 86% and 25% reduction in the tumor volume, respectively, after intravenous injection at 200 nM in the mouse model of metastatic bone tumors.

#### Antibacterial PNAs

Antisense PNAs have been extensively studied as potential antibacterial agents. The scope and limitation of these studies have been recently reviewed [318], therefore only examples where in vivo data were presented are included in Table 1. Cell-penetrating peptides (CPP) are the most commonly used ligands for delivery of PNA to bacteria; however, this approach is not universally applicable because the CPP-mediated transport across bacterial cell membrane may be dependent on the specific strain of bacteria. Tan and co-workers showed that PNA conjugated with the (KFF)_3_K peptide inhibited bacterial growth in vivo in BALB/c mice infected with SM101 or K12 strains of *E. coli* [305]. The antisense PNAs targeting the acpP gene that encodes protein ACP responsible to fatty acid biosynthesis were more effective against the SM101 strain, which has a defective outer membrane and hence is easier penetrated by PNA conjugates.

Seleem and co-workers compared conjugation to five different CPPs for delivery of PNA antisense to *rpo*A gene encoding RNA polymerase α subunit, which also causes suppressive effects on other essential bacterial genes and virulence factors [306]. In murine macrophage cells infected with *Listeria monocytogenes*, (RXR)_4_XB–PNA conjugate was the most effective, with significant reduction at 2 μM and complete clearance of intracellular *Listeria* at 8 μM. Tat–PNA and (RFR)_4_XB–PNA conjugates also showed significant activity at 2 μM. In *C. elegans* infected with *L. monocytogenes*, the (RXR)_4_XB–PNA conjugate achieved complete bacterial clearance at 32 μM. Collectively, the in vitro and in vivo results suggested that (RXR)_4_XB followed by Tat and (RFR)_4_XB were the best CPPs for delivery of the anti-*rpo*A PNA to cells infected with *L. monocytogene* [306].

While PNA–CPP conjugates have shown promising anticancer and antibacterial activity in cell cultures and in vivo, they are not without drawbacks and vulnerabilities. Activity of PNA–CPP conjugates can drastically decrease in the presence of blood serum [319] and typically require excessively high (10 to 50 mg/kg) and repeated dosing to achieve therapeutic effect in vivo [16,157]. In addition, some CPPs are larger than their PNA cargo, increasing the complexity of the therapeutic system. Despite extensive studies, primary literature lacks reports on comprehensive and conclusive studies on long-term toxicity and possible innate and adaptive immune responses [320]. In summary, while many attempts at therapeutic development have given promising preliminary results, PNAs have still not entered clinical trials [14].

## Conclusion

The year 2021 marks the 30th anniversary of the original PNA publication [1]. The remarkable biophysical properties of the first neutral DNA mimic, especially the high binding affinity and sequence selectivity for complementary native nucleic acids, were recognized immediately. However, the limitations imposed by poor solubility and inefficient crossing of cellular membranes quickly became obvious. Over these 30 years, extensive research focused on either direct chemical modification or conjugation of PNA with various ligands to address the limitations and improve the biophysical and biological properties of PNA. The present review covers only selected examples of an enormous body of these studies, but aims to present a comprehensive picture of the versatility of PNA.

It is fascinating to think that, while many chemical modifications of both backbone and nucleobases have been reported, relatively few provide significant improvements on the original design. Among the backbone modifications, pre-organizing of PNA in a right-handed helix favorable for DNA binding either by cyclopentane or γ-substituents has shown the most promise. Work towards the development of nucleobase modifications continues to address the limitations of triple helical recognition of dsDNA and dsRNA. Cellular uptake remains an unsolved problem, and both backbone and nucleobase modifications may deliver future advances. In this context, the 2-aminopyridine (M) nucleobase has afforded interesting preliminary results by enhancing both molecular recognition of dsRNA and cellular uptake of triplex-forming PNAs. In applications where solubility and cell permeability are not the limiting factors, such as PCR or FISH, PNA is widely used due to its exceptional binding strength and specificity.

Cell-penetrating peptides have been extensively explored as delivery-enhancing ligands. While many of the conjugates have shown promising in vitro and even in vivo activity, PNA-based therapeutic candidates have not yet entered clinical trials. It appears that the key remaining bottleneck is the necessity for high doses of PNA conjugates, to overcome the problem of endosomal entrapment, and associated toxicity. In other words, the chemical modifications that have succeeded in addressing the problems of cellular uptake, biodistribution, and tissue delivery of PNA have also increased the toxicity of the conjugates beyond acceptable therapeutic windows. Nevertheless, both academic and industrial research groups continue creative research into new chemical modifications and PNA–ligand conjugations. The optimism remains high, that with the right combination of innovative chemistry and biology, the full potential of PNA in biomedical applications will be discovered in the near future.
